# A Distinct Role of Riplet-Mediated K63-Linked Polyubiquitination of the RIG-I Repressor Domain in Human Antiviral Innate Immune Responses

**DOI:** 10.1371/journal.ppat.1003533

**Published:** 2013-08-08

**Authors:** Hiroyuki Oshiumi, Moeko Miyashita, Misako Matsumoto, Tsukasa Seya

**Affiliations:** Department of Microbiology and Immunology, Graduate School of Medicine, Hokkaido University, Kita-ku, Sapporo, Japan; University of Washington, United States of America

## Abstract

The innate immune system is essential for controlling viral infections, but several viruses have evolved strategies to escape innate immunity. RIG-I is a cytoplasmic viral RNA sensor that triggers the signal to induce type I interferon production in response to viral infection. RIG-I activation is regulated by the K63-linked polyubiquitin chain mediated by Riplet and TRIM25 ubiquitin ligases. TRIM25 is required for RIG-I oligomerization and interaction with the IPS-1 adaptor molecule. A knockout study revealed that Riplet was essential for RIG-I activation. However the molecular mechanism underlying RIG-I activation by Riplet remains unclear, and the functional differences between Riplet and TRIM25 are also unknown. A genetic study and a pull-down assay indicated that Riplet was dispensable for RIG-I RNA binding activity but required for TRIM25 to activate RIG-I. Mutational analysis demonstrated that Lys-788 within the RIG-I repressor domain was critical for Riplet-mediated K63-linked polyubiquitination and that Riplet was required for the release of RIG-I autorepression of its N-terminal CARDs, which leads to the association of RIG-I with TRIM25 ubiquitin ligase and TBK1 protein kinase. Our data indicate that Riplet is a prerequisite for TRIM25 to activate RIG-I signaling. We investigated the biological importance of this mechanism in human cells and found that hepatitis C virus (HCV) abrogated this mechanism. Interestingly, HCV NS3-4A proteases targeted the Riplet protein and abrogated endogenous RIG-I polyubiquitination and association with TRIM25 and TBK1, emphasizing the biological importance of this mechanism in human antiviral innate immunity. In conclusion, our results establish that Riplet-mediated K63-linked polyubiquitination released RIG-I RD autorepression, which allowed the access of positive factors to the RIG-I protein.

## Author Summary

The cytoplasmic viral RNA sensor RIG-I recognizes various types of pathogenic viruses and evokes innate immune responses, whereas several viruses have evolved strategies to escape the host innate immune responses. RIG-I triggers a signal to induce type I interferon and inflammatory cytokines. RIG-I activation is regulated by K63-linked polyubiquitin chains mediated by the ubiquitin ligases TRIM25 and Riplet; however, the functional difference between the two ubiquitin ligases remains unclear, and the molecular mechanism underlying Riplet-mediated RIG-I activation is unknown. We revealed sequential roles of the two ubiquitin ligases during RIG-I activation and found that Riplet-mediated polyubiquitination of the RIG-I repressor domain released RIG-I autorepression of its N-terminal CARDs responsible for triggering the signal, which resulted in an association with TRIM25 ubiquitin ligase and TBK1 protein kinase. Interestingly, we found that this mechanism was targeted by hepatitis C virus, which is a major cause of hepatocellular carcinoma. This result emphasizes the vital role of Riplet-mediated release of RIG-I RD autorepression in antiviral responses. Our results establish that Riplet releases RIG-I RD autorepression and demonstrated the biological significance of this mechanism in human innate immune responses.

## Introduction

The innate immune system is essential for controlling virus infections, and several viruses have evolved strategies to evade host innate immune responses. Cytoplasmic viral RNA is recognized by RIG-I-like receptors, including RIG-I and MDA5 [1], [2]. The RIG-I protein comprises N-terminal Caspase Activation and Recruitment Domains (CARDs), a central RNA helicase domain, and a C-terminal Repressor domain (RD) [3]. RD consists of C-terminal RNA binding domain (CTD) and a bridging domain between CTD and helicase [4]. RIG-I CARDs are essential for triggering the signal that induces type I interferon (IFN). In resting cells, RIG-I RD represses its CARDs signaling [3]. After viral infection, RIG-I RD recognizes 5′-triphosphate double-stranded RNA (dsRNA), which results in a conformational change in the RIG-I protein [3]. This conformational change leads to the release of RD autorepression of CARDs, after which CARDs associate with an IPS-1 adaptor molecule (also called MAVS, Cardif, and VISA) localized at the outer membrane of mitochondria [3], [5], [6], [7], [8]. IPS-1 activates downstream factors such as TBK1, IKK-ε, and NEMO [9], [10], [11]. NEMO forms a complex with TBK1 and IKK-ε and has a polyubiquitin binding region [12]. These protein kinases are essential for activating transcription factors such as IRF-3 to induce type I IFN production [13].

Several ubiquitin ligases are involved in regulating the RIG-I-dependent pathway, and RIG-I itself is regulated by ubiquitin chains [14]. Gack MU and colleagues firstly reported that TRIM25 ubiquitin ligase mediates K63-linked polyubiquitination of RIG-I N-terminal CARDs, which results in RIG-I activation [15]. Other groups also detected a RIG-I-anchored polyubiquitin chain after ligand stimulation or viral infection [16], [17]. It was recently demonstrated that an unanchored polyubiquitin chain but not ubiquitination is essential for RIG-I activation [18], [19]. However, RIG-I anchored K63-linked polyubiquitin chains are detected after viral infection [15], [20].

Another E3 ubiquitin ligase, Riplet, binds RIG-I RD and mediates the K63-linked polyubiquitination of RIG-I RD [21]. In contrast, Chen DY and colleagues reported that Riplet (also called Reul) ubiquitinated RIG-I CARDs [22]. A study that used Riplet knockout mice showed that mouse Riplet is essential for RIG-I-mediated type I IFN production in response to vesicular stomatitis virus (VSV), Flu, and Sendai virus (SeV) infections [23]. However, the functional difference between Riplet and TRIM25 remains unclear, and the molecular mechanism of how Riplet-mediated RIG-I ubiquitination activates RIG-I signaling remains unresolved.

Hepatitis C virus (HCV) is a major cause of hepatocellular carcinoma (HCC) worldwide. HCV RNA is primarily recognized by RIG-I *in vitro* and *in vivo* [24]. The HCV protease NS3-4A can suppress type I IFN production [25]. NS3-4A cleaves IPS-1 to suppress RIG-I-mediated innate immune responses [7], [26]. Human monocyte-derived dendritic cells recognize HCV RNA through Toll-like receptor 3, and NS3-4A has the ability to cleave TICAM-1, which is a solo adaptor molecule of Toll-like receptor 3 [27], [28]. In this study, we found that Riplet was another target of NS3-4A. Here, we demonstrated the molecular mechanisms of how Riplet-mediated RIG-I polyubiquitination triggered the type I IFN production signal and showed that this mechanism was targeted by HCV.

## Results

### Riplet and TRIM25 ubiquitin ligases play different roles in RIG-I activation

**Figure 1:**
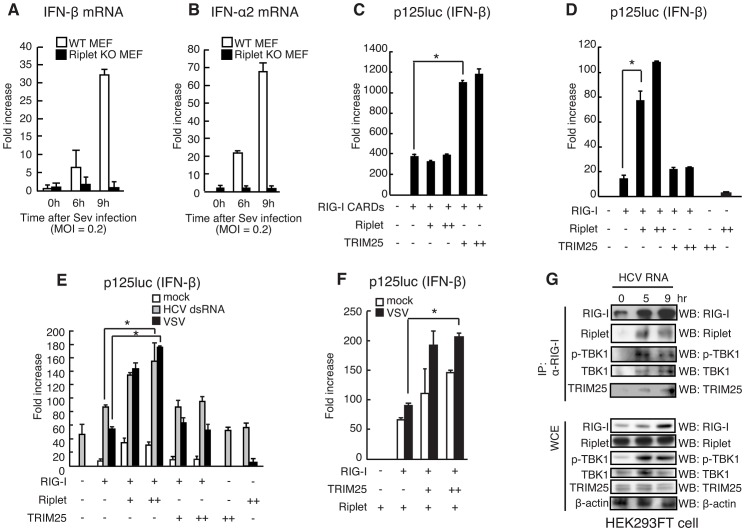
Riplet promotes TRIM25-mediated full-length RIG-I activation. (A, B) Wild-type and Riplet KO MEFs were infected with SeV at MOI = 0.2. The mRNA expressions of IFN-β (A) and –α2 (B) at the indicated times after viral infection were determined by RT-qPCR. Results are presented as mean ± SD (n = 3). (C, D) The activation of IFN-β promoter was examined by reporter gene assay using a p125luc IFN-β reporter. RIG-I CARDs (C) or full-length RIG-I (D) expression vectors were transfected into HEK293 cells together with a Riplet and/or TRIM25 expression vector as indicated. 24 hours after the transfection, the reporter activities were determined. (E, F) The activation of IFN-β promoter was examined by reporter gene assay using a p125luc IFN-β reporter. Full-length RIG-I expression vectors were transfected into HEK293 cells together with a Riplet and/or TRIM25 expression vector as indicated. 24 hours after the transfection, the cells were stimulated with 50 ng of HCV 3′ UTR dsRNA by transfection or infected with VSV at MOI = 1 for 24 hours, and then reporter activation was determined. Data are presented as mean ± SD (n = 3). *p<0.05. (G) HEK293FT cells were stimulated with 0.8 µg of HCV double-stranded RNA (HCV RNA) using lipofectamine 2000 in 6-well plate. Cell lysates were prepared at the indicated times, followed by immunoprecipitation with an anti-RIG-I mAb (Alme-1).

In mouse embryonic fibroblasts (MEFs), TRIM25 is essential for type I IFN production in response to SeV infection [15]. As with TRIM25 knockout, Riplet knockout abolished IFN-α2 and IFN-β mRNA expressions in response to SeV infection in MEF (Figure 1A and 1B), which suggested that both ubiquitin ligases were essential for type I IFN expression in MEFs in response to SeV infection. Thus, we examined whether these two ubiquitin ligases played different roles in RIG-I activation.

We performed reporter gene assays with p125luc (IFN-β reporter) using either RIG-I CARDs fragment or full-length RIG-I expression vectors. It is known that ectopic expression of RIG-I CARDs fragment activates the signaling even in the absence of stimulation with RIG-I ligand [2], [3] and that the auto-activation is observed when full-length RIG-I is ectopically expressed in HEK293 cell [21], [29]. As previously reported, TRIM25 ectopic expression efficiently increased RIG-I CARDs fragment-mediated signaling (Figure 1C). However, TRIM25 expression only mildly increased full-length RIG-I signaling (Figure 1D). In contrast, Riplet ectopic expression efficiently increased the full-length RIG-I-mediated signaling, although Riplet failed to increase the RIG-I CARDs-mediated signaling (Figure 1C and 1D). Interestingly, when Riplet was co-expressed with TRIM25, ectopically expressed TRIM25 could increase the full-length RIG-I-mediated signaling (Figure 1F). We observed the same effects of TRIM25 and Riplet expressions on the reporter activation in the presence of stimulation with HCV 3′ UTR dsRNA (a RIG-I ligand) or VSV infection, which are recognized by RIG-I (Figure 1E and 1F). These different effects of Riplet and TRIM25 on the signaling suggested that Riplet and TRIM25 ubiquitin ligases played different roles in RIG-I activation.

**Figure 2:**
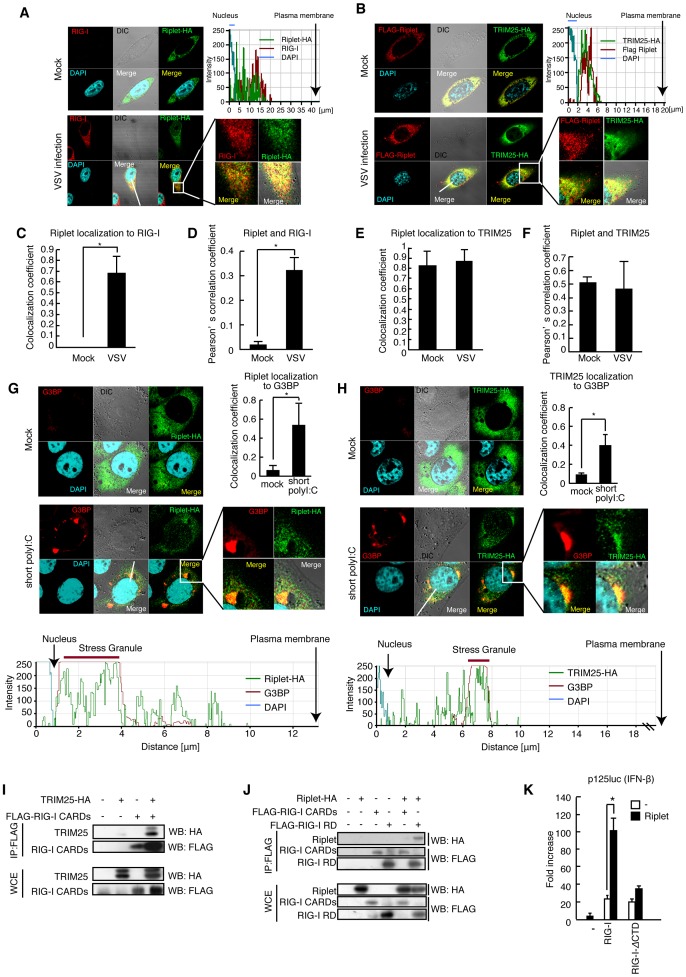
Riplet and TRIM25 ubiquitin ligases associate with RIG-I. (A, B) HeLa cells were transfected with Riplet-HA expression vector (A) or FLAG-Riplet and TRIM25-HA (B). 24 hours after transfection, the cells were infected with VSV at MOI = 1 for six hours. The cells were fixed and stained with anti-RIG-I (Alme-1), HA, and/or FLAG antibodies as indicated. Histograms display the measured fluorescence intensity along the white line in the merged panels. (C–F) Colocalization coefficients of Riplet localization to RIG-I (C) or TRIM25 (E) in mock or VSV infected HeLa cells. Pearson's correlation coefficient of Riplet and RIG-I (D) and TRIM25 (F) (mean ± SD, n>10). (G, H) HeLa cells were transfected with Riplet-HA (G) or TRIM25-HA (H) expression vector. The cells were stimulated with 100 ng of short polyI:C for six hours. The cells were fixed and stained with anti-G3BP and HA antibodies. Colocalization coefficient indicates values (mean ± SD, n>10) of Riplet (G) or TRIM25 (H) localization to G3BP staining region. Histograms display the measured fluorescence intensity along the white line in the merged panels. (I, J) TRIM25 (I) or Riplet (J) expression vector was transfected into HEK293FT cells together with FLAG-tagged-RIG-I CARDs or -RIG-I RD expression vectors. Cell lysate was prepared at 24 hours after transfection, followed by immunoprecipitation with an anti-FLAG antibody. (K) Riplet, RIG-I, and/or RIG-I-*Δ*RD, which lacks RD, expression vector was transfected into HEK293 cell with p125luc reporter. Reporter activation was determined at 24 hours after transfection. Data are presented as mean ± SD (n = 3).

We then investigated whether the two ubiquitin ligases associate with RIG-I after stimulation. First, we performed immunoprecipitation assay and found that endogenous Riplet and TRIM25 bound to endogenous RIG-I after stimulation with HCV dsRNA (Figure 1G). Second, we investigated subcellular localizations of the proteins by confocal immunofluorescence microscopy. In resting cells, RIG-I was barely detectable, whereas RIG-I exhibited punctate staining in cytoplasm after VSV infection (Figure 2A), and ectopically expressed Riplet was co-localized with RIG-I and TRIM25 in perinuclear region in VSV infected cells (Figure 2A–2C and 2E). This is consistent with a previous observation that RIG-I and TRIM25 co-localized extensively at cytoplasmic perinuclear bodies [15]. Pearson's correlation coefficient values also suggested the correlation of the colocalizations of those proteins (Figure 2D and 2F). Recently, it was reported that RIG-I recognized short polyI:C in stress granules [30]. G3BP is a marker of the stress granule [30]. In resting cells, G3BP dispersed in cytoplasm [30], and thus barely detectable (Figure 2G and 2H), whereas G3BP speckles were detected in cells stimulated with short polyI:C in the cytoplasm (Figure 2G and 2H). Riplet and TRIM25 localizations within G3BP speckles were detected in the stimulated cells (Figure 2G and 2H). Taken together, these data indicated that both Riplet and TRIM25 associated with RIG-I after stimulation.

Next, we assessed the RIG-I regions that bind to the two ubiquitin ligases using RIG-I fragments. As previously reported, TRIM25 bound to RIG-I CARDs fragment (Figure 2I). However, Riplet bound to RIG-I RD (735–925 aa), but not to CARDs fragment (Figure 2J). Deleting the Riplet binding region (RIG-I-*Δ*CTD) abrogated Riplet effect on RIG-I signaling (Figure 2K). This was contrast to TRIM25, which affects RIG-I CARDs. Taken together, our genetic and biochemical data indicated that the two ubiquitin ligases associated with RIG-I after stimulation but showed different effects on RIG-I activation. Thus, we next focused on Riplet specific role in RIG-I activation.

### Riplet-mediated RIG-I polyubiquitination is dispensable for RIG-I RNA binding activity

**Figure 3:**
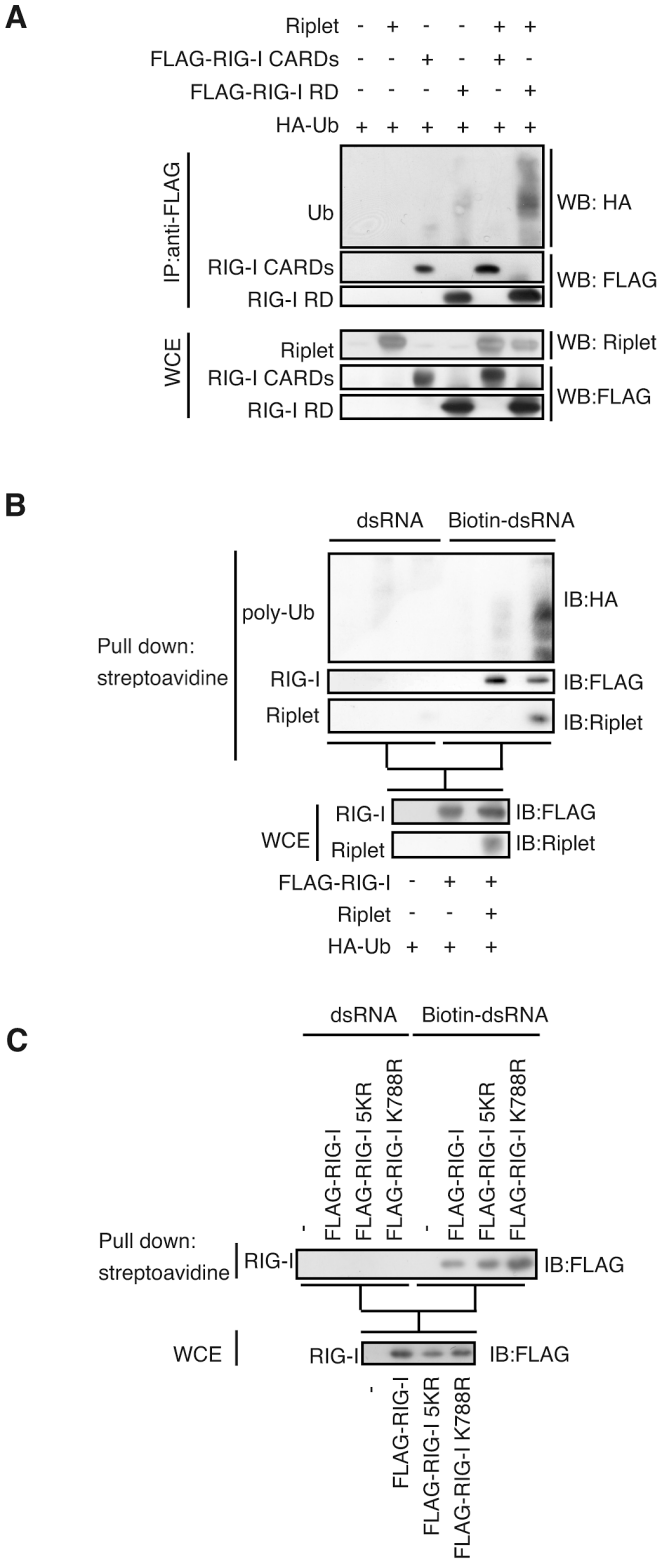
Riplet function is dispensable for RIG-I RNA binding activity. (A) Expression vectors encoding Riplet, FLAG-tagged RIG-I CARDs, and/or FLAG-tagged RIG-I RD were transfected into HEK293FT cells together with an HA-tagged ubiquitin expression vector. Cell lysate was prepared at 24 hours after transfection, followed by immunoprecipitation with an anti-FLAG (RIG-I) antibody. (B) HEK293FT cells were transfected with expression vectors encoding FLAG-tagged RIG-I, Riplet, and HA-tagged ubiquitin. Cell lysate was prepared at 24 hours after transfection, and then incubated with biotin-conjugated (Biotin-dsRNA) or non-conjugated (dsRNA) double-stranded RNA. Biotin-dsRNA was pull-downed with streptavidin beads. Samples were subjected to SDS-PAGE, and proteins were detected by western blotting. (C) HEK293FT cells were transfected with FLAG-tagged wild-type RIG-I, RIG-I 5KR, or RIG-I K788R expression vector. 24 hours after the transfection, the cell lysate was prepared. The pull down assay with biotin-dsRNA was performed as described above.

RIG-I CARDs harbor K63-linked polyubiquitination [15]. As RIG-I CARDs, RIG-I RD harbored K63-linked polyubiquitination (Supplemental Figure S1). Riplet expression increased the polyubiquitination of RIG-I RD but not that of CARDs (Figure 3A). RIG-I RD has two functions. One is RNA binding activity and the other is autorepression of its CARDs signaling. Firstly, we tested whether Riplet affects RIG-I RNA binding activity. In a pull-down assay using biotin-conjugated dsRNA and streptavidin beads, we found that both polyubiquitinated and non-ubiquitinated RIG-I were recovered (Figure 3B), which suggested that Riplet-mediated polyubiquitination was dispensable for RIG-I RNA binding activity. As RIG-I is known to form homo-oligomers [3], it is possible that non-ubiquitinated RIG-I was recovered through ubiquitinated RIG-I by the pull-down assay. To exclude this possibility, we used RIG-I mutants, RIG-I 5KR and RIG-I K788R, which were barely ubiquitinated by Riplet (described below). We compared the binding abilities of the RIG-I mutants to that of wild-type RIG-I by pull-down assay using cell lysate isolated from cells without stimulation with RIG-I ligand to avoid any ubiquitination. The results showed that the RIG-I 5KR and RIG-I K788R mutant proteins were recovered by pull down assay as wild-type RIG-I (Figure 3C). This data also indicated that the polyubiquitination is dispensable for RIG-I RNA binding activity.

### Riplet releases RIG-I RD autorepression of CARDs signaling

**Figure 4:**
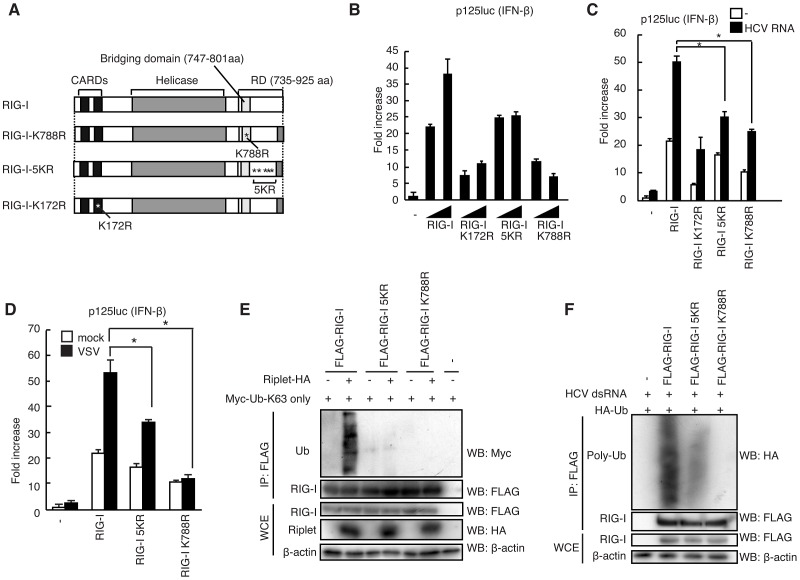
Lys residues within RIG-I RD were essential for Riplet-mediated K63-linked polyubiquitination. (A) Schematic diagram of RIG-I mutant proteins. (B–D) IFN-β promoter activation was determined using a p125luc IFN-β reporter gene. HEK293 cells were transfected with 0.1 µg of expression vectors encoding full-length RIG-I, RIG-I K172R, RIG-I 5KR, and RIG-I K788R in 24-well plate. The transfected cells were stimulated with 0.1 µg of HCV 3′ UTR dsRNA using lipofectamine 2000 reagent for eight hours (C) or infected with VSV at MOI = 1 for 24 hours (D), after which reporter activation was determined. Data are presented as mean ± SD (n = 3). *p<0.05. (E) The expression vectors encoding HA-tagged Riplet and FLAG-tagged RIG-I, RIG-I 5KR, and RIG-I K788R were transfected into HEK293FT cells together with Myc-tagged K63-only ubiquitin expression vector, in which all Lys residues except Lys-63 within ubiquitin were replaced with Arg. At 24 hours after transfection, cell lysates were prepared, followed by immunoprecipitation with an anti-FLAG (RIG-I) antibody. (F) The expression vectors encoding FLAG-tagged wild-type and mutant RIG-I proteins were transfected into HEK293FT cells together with HA-tagged ubiquitin expression vector. 24 hours after the transfection, the cells were stimulated with HCV 3′ UTR dsRNA by transfection for eight hours. Then cell lysate was prepared and immunoprecipitation was performed with anti-FLAG antibody.

Secondly, we investigated whether Riplet expression affects RIG-I RD autorepression of CARDs signaling. To test this possibility, we constructed RIG-I mutant proteins (Figure 4A). We previously showed that Lys to Ala amino acid substitutions at Lys-849, 851, 888, 907, and 909 of RIG-I (RIG-I 5KA) severely reduced RIG-I polyubiquitination and activation [21]. However, it is possible that the substitutions of Lys with Ala affect other function of RIG-I because the substitution abolishes positive charge of Lys residues. Thus, we constructed the RIG-I 5KR mutant protein, in which the five Lys residues were substituted with Arg, and examined the signal activation ability. The results showed that the 5KR mutation reduced RIG-I signaling, however residual activation of RIG-I 5KR was still detected (Figure 4B–4D). Thus, we assessed other Lys residues within RIG-I RD. Because Riplet is essential for RIG-I activation, it is expected that the Lys residues targeted by Riplet are conserved during evolution. Thus, we tested Lys residues within RIG-I RD conserved among vertebrate, and found that an RIG-I K788R mutation reduced RIG-I signaling at a level comparable to that by a K172R mutation, which abrogates TRIM25-mediated RIG-I activation [15], [19] (Figure 4B–4D). Interestingly, the 5KR and K788R mutations reduced the RIG-I polyubiquitination (Figure 4E). RIG-I polyubiquitination in cells stimulated with HCV dsRNA was also reduced by the K788R mutation (Figure 4F). Lys-788 is located within the 55-amino acid region (747–801 aa) of RIG-I essential for RD autorepression of CARDs signaling [31] (Figure 4A). It is possible that the reduction of RIG-I ubiquitination by the mutations was caused by the defect of RIG-I other functions, such as RNA binding activity. However, Figure 3C showed that the RIG-I 5KR and K788R mutant proteins efficiently bound to dsRNA as wild-type RIG-I. This data weakened the above possibility.

**Figure 5:**
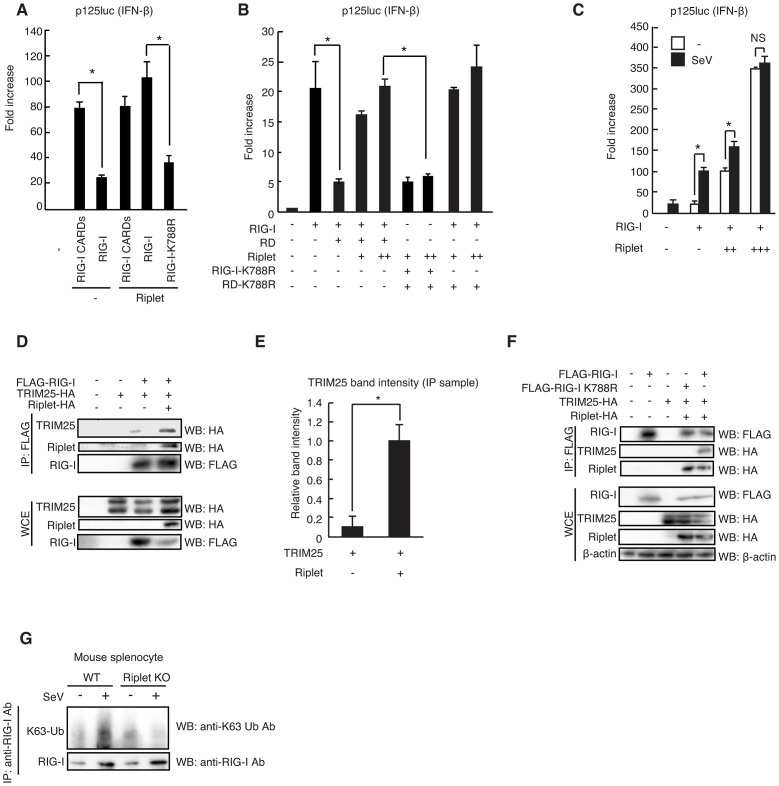
Riplet affects RIG-I RD autorepression of CARDs signaling. (A, B) IFN-β promoter activation was determined using a p125luc IFN-β reporter gene. Expression vectors encoding RIG-I CARDs, full-length RIG-I, RIG-I K788R, RD, RD-K788, and/or Riplet were transfected into HEK293 cells as indicated. Reporter activation was determined after transfection. Data are presented as mean ± SD (n = 3). *p<0.05. (C) IFN-β promoter activation was determined using a p125luc IFN-β reporter gene. RIG-I and Riplet expression vectors were transfected into HEK293 cells as indicated. 24 hours after transfection, cells were infected with SeV at MOI = 1 for 24 hours, and the reporter activities were determined. (D–F) HEK293FT cells were transfected with expression vectors encoding FLAG-tagged RIG-I, HA-tagged TRIM25, HA-tagged Riplet, FLAG-tagged RIG-I K788R as indicated. Cell lysate was prepared at 24 hours after transfection, followed by immunoprecipitation with an anti-FLAG antibody (RIG-I). Relative band intensity of immunoprecipitated TRIM25 in panel D was determined (E). (G) Mouse splenocyte was isolated from wild-type and Riplet KO mice spleen. The cells were infected with SeV, and then cell lysate was prepared. Immunoprecipitation was carried out with anti-RIG-I rabbit mAb (D14G6), and subjected to SDS-PAGE. Endogenous K63-linked polyubiquitin chain was detected using K63-linked polyubiquitin chain specific antibody.

Using the RIG-I mutants, we investigated whether Riplet releases RIG-I RD autorepression. Because Riplet ectopic expression activated full-length RIG-I signaling even in the absence of stimulation (Figure 1D), we could assess whether Riplet can remove RIG-I RD autorepression by Riplet ectopic expression study. Due to autorepression, full-length RIG-I expression weakly activates the signaling compared with RIG-I CARDs expression [31]. Riplet ectopic expression increased full-length RIG-I signaling to a level comparable to that of RIG-I CARDs signaling (Figure 5A), suggesting that Riplet released the autorepression. Moreover, the K788R substitution canceled this Riplet ectopic expression effect on RIG-I signaling (Figure 5A). This suggested that the K788R mutation abrogated the release of RIG-I RD autorepression but not RNA binding activity.

Expression of the RIG-I RD fragment is known to represses full-length RIG-I-mediated signaling [3]. Interestingly, Riplet ectopic expression removed the RD fragment repression effect on RIG-I signaling (Figure 5B). This Riplet expression effect was canceled by the K788R amino acid substitution within full-length RIG-I (Figure 5B).

MDA5 C-terminal region does not function as an RD, and thus ectopically expressed MDA5 induced IFN-β promoter activity irrespective of viral infection [3]. If Riplet ectopic expression released RIG-I RD autorepression, it is expected that RIG-I and Riplet co-expression will induce IFN-β promoter activity irrespective of viral infection. As expected, Riplet and RIG-I co-expression induced IFN-β promoter activity irrespective of SeV infection (Figure 5C). Taken together, these genetic data indicated that Riplet released RIG-I RD autorepression.

If Riplet is essential for the release of RIG-I RD autorepression, it is expected that Riplet expression will increase the interaction between RIG-I and TRIM25, because TRIM25 efficiently activated RIG-I CARDs but not full-length RIG-I (Figure 1C and 1D). To test this possibility, we examined the interaction between TRIM25 and RIG-I in the presence or absence of Riplet ectopic expression and found that Riplet expression increased the interaction between TRIM25 and RIG-I (Figure 5D and 5E). Moreover, this interaction was abolished by the K788R mutation (Figure 5F). These data were also consistent with our model that Riplet affects RIG-I RD autorepression rather than RNA binding activity of RIG-I.

If Riplet is essential for the release of RIG-I RD autorepression leading to the interaction between TRIM25 and RIG-I, it was expected that Riplet is essential for endogenous RIG-I K63-linked polyubiquitination that is mediated by both Riplet and TRIM25. To test this possibility, we investigated endogenous RIG-I K63-linked polyubiquitination in mouse spleen cells infected with SeV. Endogenous RIG-I K63-linked polyubiquitination was increased after SeV infection in wild-type splenocyte, however knockout of Riplet abrogated the endogenous K63-linked polyubiquitination of RIG-I after SeV infection (Figure 5G). Recently, it was reported that knockdown of Riplet strongly reduced endogenous RIG-I polyubiquitination in response to SeV infection in a mouse cell line Hepa 1.6 [32]. Based on our genetic and biochemical data in Figure 3–5, we concluded that Riplet affects RIG-I RD autorepression rather than the RNA binding activity.

### Riplet is required for the formation of a hetero-protein complex of RIG-I, TBK1, and IKK-ε

**Figure 6:**
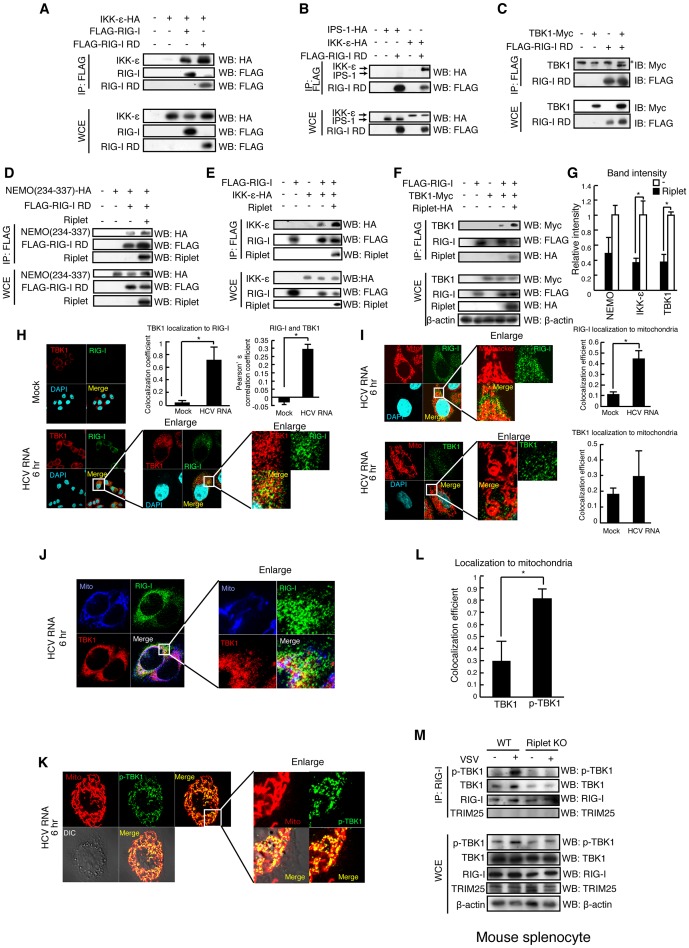
The association of TBK1 and IKK-ε protein kinases with RIG-I RD is enhanced by Riplet. (A–F) The interaction of RIG-I with TBK1, IKK-ε, and NEMO was examined by immunoprecipitation assay. FLAG-tagged RIG-I or RIG-I RD expression vector was transfected into HEK293FT cells together with HA-tagged IKK-ε (A, B, and E), Myc-tagged TBK1 (C, F), HA-tagged NEMO ubiquitin binding region (D), and/or Riplet (D–F) expression vectors as indicated. 24 hours after the transfection, cell lysate was prepared, and immunoprecipitation was performed with anti-FLAG antibody. Asterisk indicates non-specific bands. (G) Relative band intensity of immunoprecipitated NEMO, IKK-ε, and TBK1 in D–F was determined. (H–L) Intracellular localization of endogenous RIG-I (H–J), TBK1 (H–J), phosphorylated-TBK1 (p-TBK1) (K), and mitochondria (I–K) were observed using anti-RIG-I (Alme-1), TBK1, p-TBK1 mAbs, and mitotracker. HeLa cells were stimulated with HCV dsRNA for six hours using lipofectamine 2000. Colocalization coefficient of TBK1 localization to RIG-I (H), RIG-I and TBK1 localization to mitochondria (I), and TBK1 and p-TBK1 localization to mitochondria (L) were determined (mean ± sd, n>10). Person's correlation coefficient of RIG-I and TBK1 was determined (H). (M) Splenocytes from wild type and Riplet KO mouse were infected with VSV at MOI = 10 for eight hours. Immunoprecipitation was performed using an anti-RIG-I rabbit monoclonal antibody (D14G6), and the immunoprecipitates were analyzed by SDS-PAGE. Endogenous RIG-I, TBK1, TRIM25, and β-actin were detected using anti-RIG-I, p-TBK1, TRIM25, and β-actin antibodies.

Because Riplet-mediated release of RD autorepression increased the interaction between RIG-I and TRIM25, we investigated whether the release of RD autorepression also increased the interaction of RIG-I with other factors. Interestingly, we found that ectopically expressed IKK-ε, TBK1, and NEMO ubiquitin binding region co-immunoprecipitated with RIG-I RD (Figure 6A–6D), and Riplet expression enhanced the physical interactions of RIG-I with TBK1, IKK-ε, and the NEMO ubiquitin binding region (Figure 6D–6G). The physical interactions between these proteins were not through IPS-1, as IPS-1 did not co-immunoprecipitate with RIG-I RD (Figure 6B).

Microscopy analysis showed that the RIG-I was co-localized with TBK1 or NEMO in the cytoplasm. (Figures 6H and S2A). RIG-I and TBK1 was detected in the region where there is no mitochondria (Figure 6I), and the colocalization of RIG-I with TBK1 was also detected in the region where there is no mitochondria (yellow stained region in Figure 6J). This was consistent with our immunoprecipitation results that RIG-I RD could bind TBK1 without IPS-1. TBK1 is phosphorylated in its activation loop [33]. Surprisingly, the phosphorylated TBK1 (p-TBK1) foci were exclusively localized on mitochondria (Figure 6K and 6L). Co-localization of RIG-I with p-TBK1 was observed after dsRNA stimulation (Figure S2B and S2C). We next assessed the role of endogenous Riplet in the interaction between RIG-I and TBK1. Immunoprecipitation assay showed that Riplet KO reduced the interaction between endogenous RIG-I and TBK1 in mouse spleen cells during VSV infection, indicating that Riplet promoted the interaction between RIG-I and TBK1 (Figure 6M). These data indicated that the Riplet function increased the interaction of RIG-I with TBK1 as well as TRIM25.

### Hepatitis C virus protease NS3-4A targets the Riplet protein

Several viruses have evolved strategies to escape the innate immunity. For instance, NS1 of influenza A virus inhibits TRIM25 function. This emphasizes the vital role of TRIM25 in modulating antiviral response [34]. To assess the biological significance of Riplet-mediated release of RIG-I RD autorepression in antiviral innate immune response, we investigated whether viral protein suppresses this mechanism.

**Figure 7:**
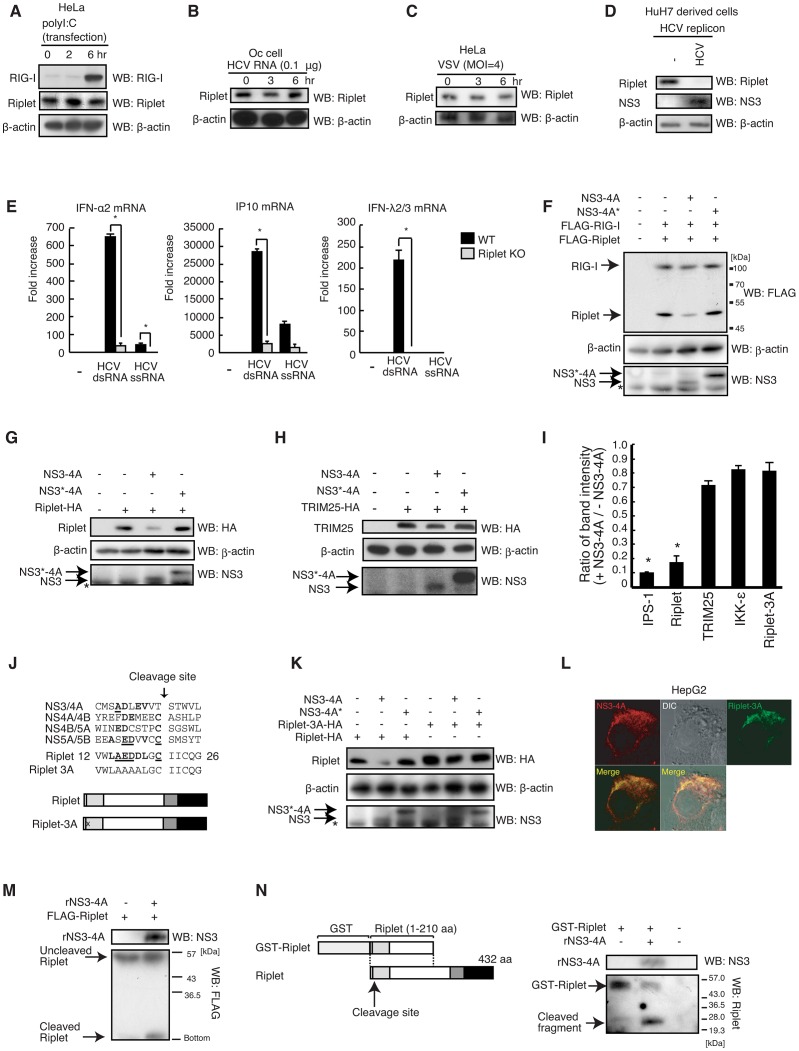
NS3-4A of HCV targets the Riplet protein. (A–D) Endogenous RIG-I and Riplet protein levels were observed by western blotting. HeLa cells were stimulated with polyI:C transfection (A), HCV dsRNA transfection (B) or infected with VSV (C). HCV replicon positive (HCV) and negative (-) cell lysates were prepared from a HuH7-derived cell line O cell that contains HCV 1b full-length replicons and O curred cell (Oc cell) in which HCV replicons were removed by IFN treatment (D). (E) The response to HCV RNA in wild-type and Riplet KO MEFs was examined by RT-qPCR. Wild type (WT) and Riplet knockout (KO) MEF cells were transfected with 100 ng of HCV ssRNA and dsRNA. Six hours after stimulation, mRNA expressions of IFN-α2, IP10, and IFN-λ2/3 were measured by RT-qPCR. Data are presented as mean ± SD (n = 3). *p<0.05. (F–H) FLAG-tagged Riplet and RIG-I (F), HA-tagged Riplet (G), or HA-tagged TRIM25 (H) expression vectors were transfected into HEK293FT cells together with NS3-4A or NS3-4A* expression vectors. NS3-4A* mutant protein harbors an amino acids substitution at its catalytic site Ser-139 with Ala. 24 hours after transfection, cell lysate was prepared and subjected to SDS-PAGE. (I) Band intensity ratio of IPS-1, Riplet, TRIM25, IKK-ε, and Riplet-3A with/without NS3-4A expression (mean ± sd, n = 3). (J) NS3-4A cleavage sites within an HCV polypeptide are compared with a candidate site in the Riplet RING-finger domain. Homologous amino acids are shown in bold, and identical amino acids are underlined. In Riplet-3A mutant protein, three acidic amino acids, Glu-16, Asp-17, Asp-18, were substituted with Ala. (K) An expression vector encoding wild-type Riplet or Ripled-3A mutant protein was transfected into HEK293 cells together with NS3-4A or NS3-4A* expression vectors. Cell lysate was prepared 24 hours after transfection, and subjected to SDS-PAGE. (L) HA-tagged Riplet-3A and NS3-4A expression vectors were transfected into HepG2 cell. 24 hours after transfection, the cells were fixed and stained with anti-HA monoclonal antibody (mouse) and anti-NS3-4A polyclonal antibody (goat). (M) N-terminal FLAG-tagged Riplet was expressed in HEK293FT cells, and immunoprecipitation was carried out with anti-FLAG antibody. Immunoprecipitates were incubated with recombination NS3-4A purified from *E.coli* at 37°C for one hour, and samples were subjected to SDS-PAGE analysis. The proteins were detected by western blotting. (N) Purified GST fused Riplet (1–210 aa) was incubated with or without recombinant NS3-4A (rNS3-4A) at 37°C for 30 min. The proteins were subjected to SDS-PAGE and detected by western blotting.

The endogenous Riplet protein level was not affected by polyI:C, HCV dsRNA stimulations, or by VSV infection (Figure 7A–7C), however the Riplet protein level was severely reduced in a human hepatocyte cell line with HCV 1b full-length replicons (O cells) compared with hepatocyte cell line without these replicons (O curred cells: Oc cells; Figure 7D), suggesting that viral protein reduced the Riplet protein level. Riplet knockout abolished the expression of type I IFN, IP-10, and type III IFN in response to HCV RNA (Figure 7E), indicating that Riplet was essential for type I IFNs expression in response to HCV RNA.

Because HCV protease NS3-4A suppresses type I IFN expression in response to viral infection [7], [25], we examined whether NS3-4A could cleave the Riplet protein. N-terminal FLAG-tagged Riplet or C-terminal HA-tagged Riplet was expressed with or without NS3-4A, after which the Riplet protein levels were compared. NS3-4A expression severely reduced the FLAG-tagged and HA-tagged Riplet protein level but not that of FLAG-tagged RIG-I, whereas catalytically inactive NS3-4A* (S139A) failed to reduce the Riplet protein level (Figure 7F, 7G, and S3A). This suggested that this protease's activity reduced the Riplet protein level. Although NS3-4A reduced IPS-1 protein level as previously reported, NS3-4A did not reduce the TRIM25 and IKK-ε protein levels (Figure 7H, 7I, S3B–D).

Within the Riplet RING-finger domain is a sequence that is similar to the NS3-4A target consensus sequence (D/E-x-x-x-x-C/T-S/A; Figure 7J) [35]. NS3-4A cleaves the target just after C/T within this consensus sequence. Acidic amino acids before the C/T site are conserved among the NS3-4A cleavage site within HCV polypeptide. The acidic amino acids from 16 to 18 aa within the Riplet sequence were substituted with Ala, and a Riplet-3A mutant protein was constructed (Figure 7J). The Riplet-3A mutant protein was resistant to NS3-4A (Figure 7I and 7K). Moreover, the Riplet-3A protein co-localized with NS3-4A in cytoplasm (Figure 7L). Interestingly, recombinant NS3-4A that was purified from *E. coli* cleaved the immunoprecipitated FLAG-tagged Riplet protein and recombinant GST-fused Riplet protein purified from *E.coli*, and the cleaved fragments were detected at expected size (Figure 7M and 7N). These data indicated that NS3-4A directly targeted the Riplet protein.

Although Riplet digestion products were not observed in HEK293 cell lysate (Figure S3E and S3F), it is known that the digestion products of TICAM-1 (TRIF) obtained by NS3-4A are not detectable because these products are unstable [28]. Cys-21 of Riplet corresponds to the C/T site in the NS3-4A target consensus sequence. The Cys-21 residue is the first Cys in the RING-finger motif; thus a C21A substitution causes the disruption of RING finger domain structure [36]. The Riplet-C21A mutant protein was unstable and barely detectable (Figure S3G), which suggested that the loss of Cys-21 destabilized the Riplet protein.

### HCV protease NS3-4A abolishes an early step of RIG-I activation

**Figure 8:**
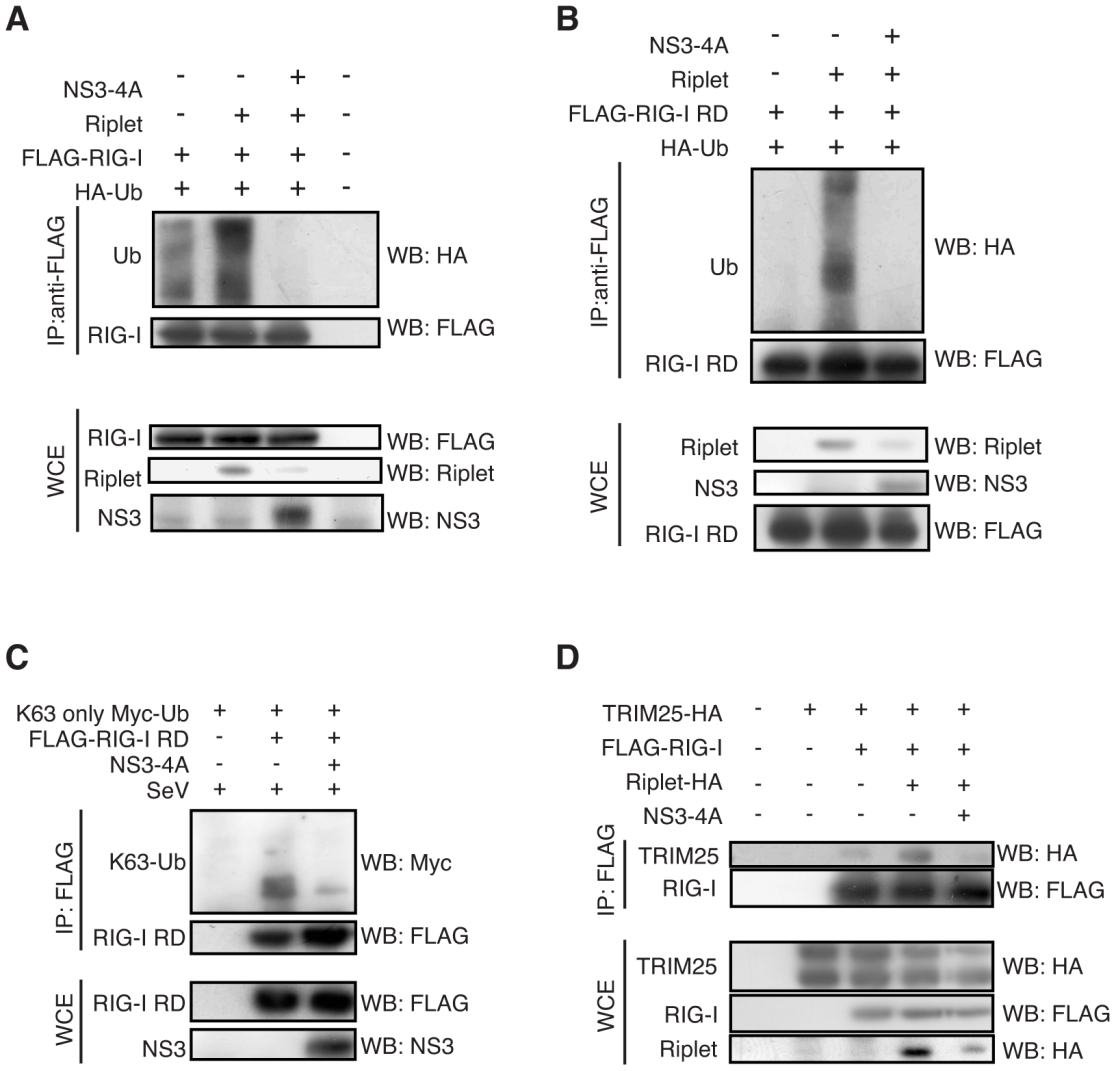
NS3-4A inhibits Riplet-mediated RIG-I polyubiquitination. (A, B) Riplet, NS3-4A, and/or HA-tagged ubiquitin (HA-Ub) expression vectors were transfected into HEK293FT cells along with either full-length RIG-I (A) or RIG-I RD (B). Cell lysate was prepared 24 hours after transfection, and subjected to SDS-PAGE. The proteins were detected by western blotting. (C). HEK293FT cells were transfected with Myc-tagged K63-only ubiquitin, FLAG-tagged RIG-I RD, and/or NS3-4A expression vectors. 24 hours after the transfection, cells were infected with SeV for six hours, and then cell lysate was prepared. Immunoprecipitation was carried out with anti-FLAG antibody, and the samples were subjected to SDS-PAGE. (D) HA-tagged TRIM25, Riplet and/or FLAG-tagged RIG-I expression vectors were transfected into HEK293FT cells with or without NS3-4A expression vector. Cell lysate was prepared 24 hours after the transfection, and immunoprecipitation assay was performed with anti-FLAG antibody. The precipitates were subjected to SDS-PAGE.

To determine if NS3-4A abolishes RIG-I activation by disrupting Riplet, we examined RIG-I ubiquitination and interaction between RIG-I and TRIM25. Riplet-mediated ubiquitination of full-length RIG-I or RIG-I RD was abolished by NS3-4A expression (Figure 8A and 8B). K63-linked polyubiquitination of RIG-I RD in SeV infected cells were also reduced by NS3-4A expression (Figure 8C). Moreover, NS3-4A expression reduced the interaction between TRIM25 and full-length RIG-I (Figure 8D).

**Figure 9:**
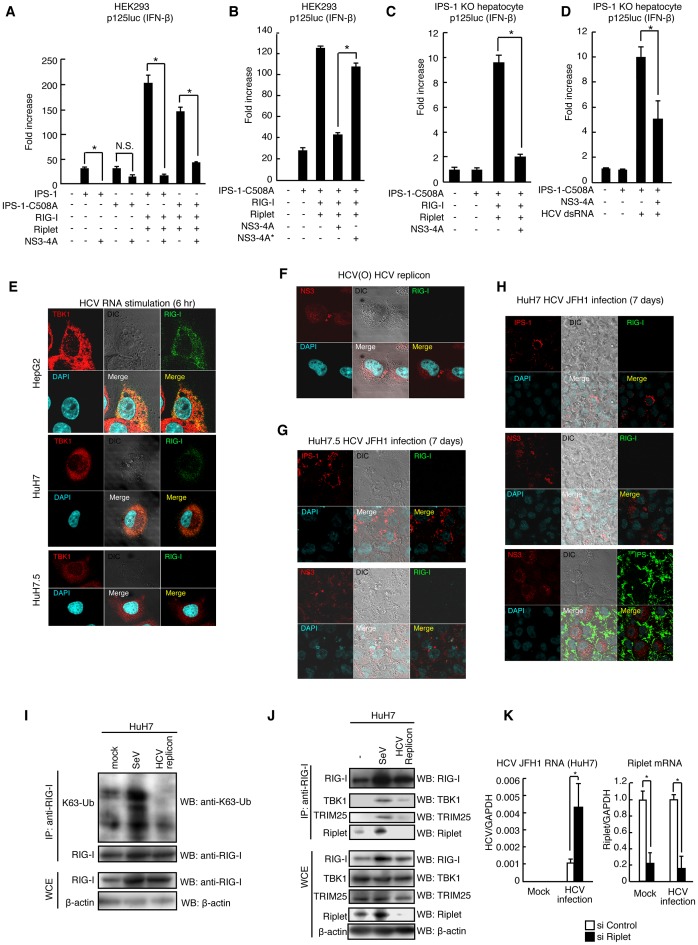
HCV abrogated Riplet-mediated RIG-I activation. (A and B) The inhibition of IFN-β promoter activation by NS3-4A was assessed by reporter gene assays. IPS-1-C508A mutant protein harbors an amino acid substitution at Cys-508 with Ala. 100 ng of IPS-1, IPS-1-C508A, RIG-I, Riplet, NS3-4A, and/or NS3-4A* expression vectors were transfected into HEK293 cells in 24-well plates with p125luc reporter plasmid. The total amount of transfected DNA (800 ng/well) was kept constant by adding empty vector (pEF-BOS). 24 hours after the transfection, the reporter activities were measured. Data are presented as mean ± SD (n = 3). *p<0.05. (C and D) IPS-1 KO mouse hepatocyte was transfected with IPS-1 C508A, RIG-I, Riplet, and/or NS3-4A expression vectors together with p125luc and *Renilla* luciferase plasmids. Transfected cells were stimulated with 50 ng of HCV dsRNA for 24 hours by transfection (D). Data are presented as mean SD (n = 3). *p<0.05. (E and F) Intracellular localizations of endogenous TBK1 and RIG-I were determined by confocal microscopy. HepG2, HuH7, and HuH7.5 cells were stimulated with 100 ng of HCV dsRNA for six hours by transfection (E). Stimulated cells (E) and O cells with HCV replicons (F) were stained with anti-RIG-I, TBK1, and/or NS3 antibodies. (G and H) HuH7 (G) and HuH7.5 (H) cells were infected with HCV JFH1 strain. Seven days after the infection, the cells were stained with anti-RIG-I, IPS-1, and NS3 antibodies. (I) HuH7 cells were infected with SeV at MOI = 1 for 24 hours. Cell lysates were prepared from mock or SeV infected HuH7 or HuH7 cells with HCV replicons (O cell). Immunoprecipitation using high salt buffer was performed with anti-RIG-I (Alme-1) antibody. The samples were subjected to SDS-PAGE. Endogenous K63-linked polyubiquitin chain was detected using ubiquitin K63-linkage specific antibody. (J) HuH7 cells were infected with SeV at MOI = 1 for 24 hours. Cell lysates were prepared from mock or SeV infected HuH7 or HuH7 cells with HCV replicons (O cell). Immunoprecipitation was performed with anti-RIG-I (Alme-1) antibody. The samples were subjected to SDS-PAGE. (K) HuH7 cells were transfected with siRNA for mock or Riplet. 48 hours after the transfection, cells were infected with HCV JFH1 for 2 days. RT-qPCR was performed to determine HCV genome RNA, GAPDH, and Riplet expression.

An IPS-1-C508A mutant protein is resistant to NS3-4A cleavage [7]. A reporter gene assay showed that NS3-4A expression severely reduced IFN-β promoter activation induced by RIG-I and Riplet expression even in the presence of the IPS-1 C508A mutant protein in HEK293 cell (Figure 9A), and a catalytically inactive NS3-4A mutant failed to reduce this signal (Figure 9B). These data were consistent with our observation that NS3-4A targeted the Riplet protein. There was endogenous IPS-1 in HEK293 cell, and thus the decrease of IFN-β promoter induction by NS3-4A in HEK293 cell could be due to the cleavage of endogenous IPS-1 by NS3-4A. To exclude this possibility, we next used IPS-1 KO mouse hepatocyte [37]. The IPS-1 C508A mutant protein was expressed in IPS-1 KO hepatocyte. NS3-4A expression reduced the reporter activation induced by RIG-I/Riplet expression or by stimulation with HCV dsRNA even in the presence of IPS-1 C508A (Figure 9C and 9D). These results indicated that NS3-4A targeted both IPS-1 and upstream factors of IPS-1 and were consistent with our observation that NS3-4A reduced the Riplet protein level. We could not test whether NS3-4A failed to impair IFN-β promoter activity in the presence of the Riplet-3A and Riplet C21A mutants because the mutant proteins were not functional and failed to activate RIG-I (Supplemental Figure S3H and S3I). Thus, we could not exclude the possibility that NS3-4A targeted another protein in addition to Riplet and IPS-1.

### Hepatitis C virus abrogates endogenous Riplet function

Endogenous RIG-I exhibited punctate staining in the human hepatocyte cell line HuH7 and HepG2 cells after simulation with HCV RNA (Figure 9E) but not in HuH7.5 cell line (Figure 9E). In HuH7.5 cells, there is a T55I mutation within endogenous *RIG-I* gene that disrupts the interaction between RIG-I and TRIM25 [38]. We investigated whether Riplet is required for RIG-I to exhibit punctate staining, and found that knockdown of Riplet decreased RIG-I punctate staining induced by HCV RNA (Supplemental Figure S4). We next investigated whether HCV abrogated Riplet-dependent RIG-I punctate pattern in the cytoplasm. As expected, RIG-I failed to exhibit punctate staining in O cells with HCV replicons in NS3 positive cells and HuH7 cells infected with HCV JFH1 (Figure 9F–9H). We confirmed that HCV disrupted IPS-1 in our experimental condition (Figure 9G and 9H).

To further assess whether HCV abrogates endogenous Riplet function, we observed endogenous K63-linked polyubiquitination of RIG-I in cells with HCV replicons. Although SeV infection induced endogenous K63-linked polyubiquitination of RIG-I in HuH7 cells, HCV replicons failed to increase the polyubiquitination (Figure 9I). Next, we investigated the association of endogenous RIG-I with TRIM25 and TBK1, which is promoted by Riplet as shown in Figure 5 and 6. SeV infection induced the association of endogenous RIG-I with TRIM25 and TBK1, whereas HCV replicons failed to induce the association (Figure 9J). Taken together, these data indicated that HCV abrogated endogenous Riplet function.

Although NS3-4A cleaves IPS-1, a mutation within endogenous *RIG-I* gene increased the permissiveness to HCV infection in HuH7-derived cells [3], indicating that RIG-I is required for antiviral response to HCV infection before NS3-4A cleaves IPS-1. We used siRNA to knockdown endogenous Riplet in HuH7 cells, and then the cells were infected with HCV JFH1. Interestingly, Riplet knockdown increased the permissiveness to HCV JFH1 infection (Figure 9K), indicating that endogenous Riplet is required for antiviral response to HCV infection.

## Discussion

RIG-I activation is regulated by two ubiquitin ligases Riplet and TRIM25 [15], [21]. The two ubiquitin ligases are essential for RIG-I activation [15], [23], however the functional difference had been unclear. It is known that TRIM25 is essential for RIG-I oligomerization and association with IPS-1 adaptor molecule [15], [19]. Here, we demonstrated that Riplet was essential for the release of RIG-I RD autorepression of its CARDs, which resulted in the association with TRIM25. This functional difference explained the reason why RIG-I requires the two ubiquitin ligases for triggering the signal.

**Figure 10:**
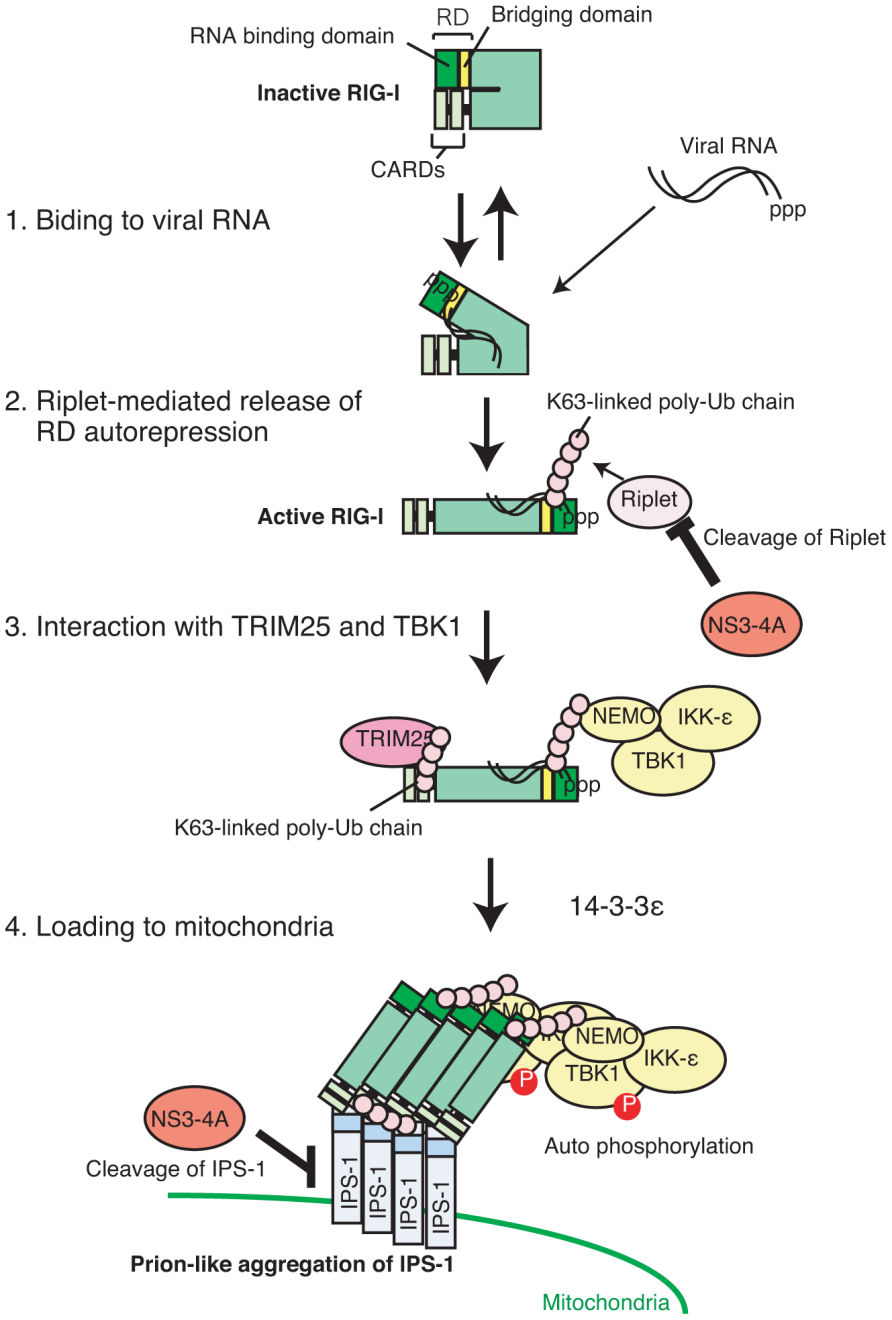
Model for Riplet-mediated RIG-I activation. In resting cell, RIG-I RD represses its CARDs-mediated signaling. When RIG-I CTD associates with viral RNA, Riplet mediates K63-linked polyubiquitination of RIG-I RD, leading to the association with TRIM25 and TBK1. K63-linked polyubiquitin chain mediated by TRIM25 induces RIG-I oligomerization and association with IPS-1 adaptor. TBK1 associated with RIG-I is activated on mitochondria.

It has been reported that TRIM25 activates RIG-I signaling [15], [19]. We confirmed that ectopic expression of TRIM25 increases RIG-I CARDs-mediated signaling. However, most previous studies used a RIG-I CARDs fragment but not full-length RIG-I [15], [19], [38]. Unexpectedly, we found that the increase of full-length RIG-I-mediated signaling by TRIM25 expression was much less than that of the CARDs-mediated signaling. It is intriguing that Riplet helped TRIM25 to activate full-length RIG-I. Riplet expression promoted the interaction between TRIM25 and full-length RIG-I, and this interaction was abrogated by an RIG-I K788R mutation, which reduced Riplet-mediated RIG-I ubiquitination. Thus, we propose that Riplet-mediated polyubiquitination of RIG-I RD is a prerequisite for TRIM25 to activate RIG-I (Figure 10).

Ectopic expression of Riplet activated RIG-I without stimulation with RIG-I ligand. This is not surprising because ectopically expressed Riplet bound to RIG-I without stimulation with RIG-I ligand, whereas endogenous Riplet bound to endogenous RIG-I after stimulation with RIG-I ligand. RIG-I undergoes its conformational change after binding to a ligand [3], [39]. The conformational change would allow the access of endogenous Riplet to RIG-I, which resulted in Riplet-mediated K63-linked polyubiquitination leading to the release of RD autorepression. This model is consistent with the observation that TRIM25 ectopic expression did not activate full-length RIG-I without Riplet expression, because TRIM25 hardly bound to full-length RIG-I without Riplet.

Previously, we reported that the five Lys residues within RIG-I RD were important for Riplet-mediated RIG-I ubiquitination. We constructed the RIG-I 5KR mutant and indicated that the 5KR mutation reduced RIG-I ubiquitination and activation without loss of RNA binding activity. This is consistent with our previous conclusion. However, there is residual ubiquitination of RIG-I 5KR mutation, and we found that K788R mutation showed more sever phenotype. These data indicated that Riplet targeted the several Lys residues within RIG-I RD. This is not surprising, because TRIM25 targets not only Lys-172 but also other Lys residues within mouse RIG-I CARDs [32].

TBK1 and IKK-ε are downstream factors of IPS-1. We found that TBK1 and IKK-ε could bind RIG-I RD. It is possible that RIG-I associates with TBK1 through IPS-1. However, Hiscott J and colleagues demonstrated that IKK-ε could bind IPS-1 and that TBK1 did not bind IPS-1 [40]. Moreover, RIG-I RD did not bind IPS-1, and RIG-I and TBK1 co-localization was detected in the cytoplasmic region where there are no mitochondria. These observations weaken this possibility. Our results indicated that RIG-I RD bound to the NEMO ubiquitin binding region. IRF-3 activation requires the ubiquitin binding domain of NEMO, and an endogenous K63-linked polyubiquitin chain plays a key role in IRF3 activation [41]. Thus, we prefer a model in which TBK1 associates with an RIG-I RD-anchored polyubiquitin chain through NEMO (Figure 10). Although Riplet knockout reduced the binding of RIG-I to TBK1, residual binding was still detectable. Thus, there appears to be Riplet-dependent and independent associations between RIG-I and TBK1. TRAF3 is an E3 ubiquitin ligase, and is involved in the RIG-I-mediated type I IFN production pathway [42]. Because there is residual activation of the type I IFN production pathway even in TRAF3 knockout cells [41], it is possible that the RIG-I polyubiquitin chain may compensate for the TRAF3 defect in recruiting TBK1 to mitochondria. Further studies will be needed to determine the precise molecular mechanisms. Although TBK1 dispersed in the cytoplasm, p-TBK1 was exclusively localized on mitochondria. Considering that TBK1 is phosphorylated in its activation loop [33], these results suggested that RIG-I RD associated with inactive TBK1 and that TBK1 was activated after loading on to mitochondria (Figure 10).

HCV is a major cause of HCC and has the ability to evade host innate immune response [7], [43]. HCV RNA is primarily recognized by the cytoplasmic viral RNA sensor RIG-I. Previous studies showed that the protease NS3-4A cleaves IPS-1 to shut off RIG-I signaling. However, our results indicated that there was another target of NS3-4A in RIG-I signaling. First, RIG-I failed to exhibit punctate staining in cells infected with HCV. Second, NS3-4A reduced RIG-I signaling even in the presence of an IPS-1-C508A mutant, which is resistant to the cleavage by NS3-4A. Third, the endogenous Riplet protein level was severely reduced in cells with HCV replicons. Fourth, NS3-4A targeted Riplet and abrogated Riplet-dependent RIG-I ubiquitination and complex formation with TRIM25 and TBK1. These data support our model that NS3-4A targets not only IPS-1 but also Riplet to escape host innate immune responses (Figure 10). Recently it was reported that NS1 proteins of Influenza A virus inhibited Riplet function [32]. These findings indicated biological importance of Riplet in RIG-I activation during viral infection.

In general, a ubiquitin ligase has several targets. We have performed yeast two-hybrid screening using Riplet as bait and found a candidate clone that encodes a tumor suppressor gene. Our pilot study showed that Riplet mediated K63-linked polyubiquitination of this tumor suppressor and suppressed retinoblastoma (Rb) activity. Thus, Riplet disruption by NS3-4A might be a cause of liver disease induced by HCV infection.

## Materials and Methods

### Ethics statement

All animal studies were carried out in strict accordance with Guidelines for Animal Experimentation of the Japanese Associations for Laboratory Animal Science. The protocols were approved by the Animal Care and Use Committee of Hokkaido University, Japan (Permit Number: 08-0245 and 09-0215).

### Cell

HEK293, Vero, and HepG2 cells were cultured in Dulbecco's modified Eagle's medium low glucose medium (D-MEM) with 10% heat-inactivated fetal calf serum (FCS) (Invitrogen). HeLa cells were cultured in minimum Eagle's medium with 2 mM L-glutamine and 10% heat-inactivated FCS. HEK293FT cells were maintained in D-MEM high glucose medium containing 10% of heat-inactivated FCS (Invitrogen). Human hepatocyte cell line with HCV 1b full-length replicons (O cells) and O curred cells (Oc cells) were kindly gifted from Kato N [44]. O cells were cultured in D-MEM high glucose with 10% of heat-inactivated FCS, G418, NEAA, and L-Gln.

### Viruses

VSV Indiana strain and SeV HVJ strain were amplified using Vero cells. To determine the virus titer, we performed plaque assay using Vero cells. HCV JFH1 was amplified using HuH7.5 cells.

### Mice

Generation of IPS-1 KO and Riplet KO mice were described previously [23], [45]. Splenocyte was isolated from C57BL/6 wild-type and Riplet KO mice. Isolated cells were cultured in RPMI1640 containing 10% of heat-inactivated FCS. The preparations of wild-type and Riplet KO MEFs were described previously [23]. Preparation of IPS-1 KO mouse hepatocyte was described previously [37]. All mice were maintained under specific-pathogen free conditions in the animal facility of the Hokkaido University Graduate School of Science (Japan).

### Plasmids

Expression vectors encoding for N-terminal FLAG-tagged RIG-I, N-terminal FLAG-tagged RIG-I CARDs (dRIG-I), FLAG-tagged RIG-I *Δ*RD (RIG-I-dRD), FLAG-tagged RIG-I RD, C-terminal HA-tagged TRIM25, C-terminal HA-tagged Riplet, and Riplet-*Δ*RING (Riplet-DN) plasmids were described previously [21], [23]. The amino acids substitutions from 16 to 18 with Ala was carried out by PCR-mediated mutagenesis using primers, Ripelt-3A-F and Riplet-3A-R and pEF-BOS/Riplet plasmid as a template. The primer sequence is Riplet-3A-F: TTC CCG TGT GGC TGG CCG CGG CCG CCC TCG GCT GCA TCA TCT GCC, and Riplet-3A-R: GGC AGA TGA TGC AGC CGA GGG CGG CCG CGG CCA GCC ACA CGG GAA. RIG-I K172R and RIG-I K788R expression vectors was constructed by PCR-mediated mutagenesis using primers, RIG-I K172R-F, RIG-I K172R-R, RIG-I K788R-F and RIG-I-K788R-R, and pEF-BOS/FLAG-RIG-I plasmid as a template. The primer sequences are RIG-I K172R-F: GGA AAA CTG GCC CAA AAC TTT GAG ACT TGC TTT GGA GAA AG, RIG-I K172R-R: CTT TCT CCA AAG CAA GTC TCA AAG TTT TGG GCC AGT TTT CC, RIG-I-K788R-F: TGC ATA TAC AGA CTC ATG AAA GAT TCA TCA GAG ATA GTC AAG AA, and RIG-I-K788-R: CTT GAC TAT CTC TGA TGA ATC TTT CAT GAG TCT GTA TAT GCA G. RIG-I 5KR expression vectors were constructed by PCR-mediated mutagenesis using primers, RIG-I 849 851 RR-F, RIG-I 849 851 RR-R, RIG-I 888R-F, RIG-I 888R-R, RIG-I 907 909 RR-F, RIG-I 907 909 RR-R, and pEF-BOS/FLAG-RIG-I plasmid as a template. The primer sequences are RIG-I 849 851 RR-F: AGT AGA CCA CAT CCC AGG CCA AGG CAG TTT TCA AGT TTT G, RIG-I 849 851 RR-R: CAA AAC TTG AAA ACT GCC TTG GCC TGG GAT GTG GTC TAC T, RIG-I 888R-F: GAC ATT TGA GAT TCC AGT TAT AAG AAT TGA AAG TTT TGT GGT GGA GG, RIG-I 888R: CCT CCA CCA CAA AAC TTT CAA TTC TTA TAA CTG GAA TCT CAA ATG TC, RIG-I 907 909RR-F: GTT CAG ACA CTG TAC TCG AGG TGG AGG GAC TTT CAT TTT GAG AAG, RIG-I 907 909RR-R: CTT CTC AAA ATG AAA GTC CCT CCA CCT CGA GTA CAG TGT CTG AAC. HCV cDNA fragment encoding NS3-4A of JFH1 strain was cloned into pCDNA3.1 (-) vector. The mutation on catalytic site of NS3-4A S139A was constructed by PCR-mediated mutagenesis using primers, NS3-4A S139A-F and NS3-4A S139A-R, and pCDNA3.1 (-)/NS3-4A plasmid as a template. The primer sequences are NS3-4A S139A-F: TTC GAC CTT GAA GGG GTC CGC GGG GGG ACC GGT GCT TTG C and NS3-4A S139A-R: AAG CAC CGG TCC CCC CGC GGA CCC CTT CAA GGT CGA AAG G.

### RT-PCR and Real-Time PCR

Total RNA was extracted with TRIZOL (Invitrogen), after which the samples were treated with DNaseI to remove DNA contamination. Reverse transcription was performed using High Capacity cDNA Reverse Transcription Kit (ABI). Quantitative PCR analysis was performed using Step One software ve2.0. (ABI) with SYBER Green Master Mix (ABI). HCV ss and dsRNA was in vitro synthesized with SP6 and/or T7 RNA polymerase using 3′ UTR of HCV cDNA as template as described previously [46].

### Confocal microscopy

Cells were plated onto microscope cover glasses (matsunami) in a 24-well plate. The cells were fixed for 30 min using 3% formaldehyde in PBS and permeabilized with 0.2% Triton X-100 for 15 min. Fixed cells were blocked with 1% bovine serum albumin in PBS for 10 min and labeled with the indicated primary Abs for 60 min at room temperature. Alexa-conjugated secondary Abs were incubated for 30 min at room temperature to visualize staining of the primary Ab staining. Samples were mounted on glass slides using Prolong Gold (Invitrogen). Cells were visualized at a magnification of ×63 with an LSM510 META microscope (Zeiss). Data collected with confocal microscopy were analyzed with ZEISS LSM Image Examiner software. NS3, RIG-I, TBK1, IPS-1, and p-TBK1 were stained with anti-NS3 goat pAb (abcam), anti-RIG-I mouse mAb (Alme-1, ALEXIS BIOCHEMICALS), anti-NAK (TBK1) rabbit mAb (EP611Y, abcam), anti-MAVS (IPS-1) rabbit pAb (Bethyl Laboratories Inc), and anti-p-TBK1 rabbit mAb (Cell Signaling Technology),

### Reporter gene analysis

HEK293 cells were transiently transfected in 24-well plates using FuGene HD (Promega) or lipofectamine 2000 (Invitrogen) with expression vectors, reporter plasmids (IFN-β: p125luc), and an internal control plasmid coding *Renilla* luciferase. The total amounts of plasmids were normalized using an empty vector. Cells were lysed in a lysis buffer (Promega), and luciferase and *Renilla* luciferase activities were determined using a dual luciferase assay kit (Promega). Relative luciferase activities were calculated by normalizing the luciferase activity by control. HCV dsRNA (3′ UTR polyU/UC region) was synthesized using T7 and SP6 RNA polymerase as described previously [46].

### Pull-down assay

RNA used for the assay was purchased from JBioS. The RNA sequences are as follows: (sense strand) AAA CUG AAA GGG AGA AGU GAA AGU G; and (antisense strand) CAC UUU CAC UUC UCC CUU UCA GUU U . Biotin was conjugated at the U residue at the 3′-end of the antisense strand (underlined). Biotinylated dsRNA was phosphorylated by T4 polynucleotide kinase (TAKARA). dsRNA was incubated for one hour at 25°C with 10 µg of protein from the cytoplasmic fraction of cells that were transfected with Flag-tagged RIG-I, Riplet, and/or HA-tagged ubiquitin expressing vectors. This mixture was added into 400 µl of lysis buffer (20 mM Tris-HCl pH 7.5, 150 mM NaCl, 1 mM EDTA, 10% Glycerol, 1% NP-40, 30 mM NaF, 5 mM Na_3_VO_4_, 20 mM iodoacetamide, and 2 mM PMSF) containing 25 µl of streptavidine Sepharose beads, rocked at 4°C for two hours, harvested by centrifugation, washed three times with lysis buffer, and resuspended in SDS sample buffer.

### Immunoprecipitation

Splenocytes (1×10^7^) were infected with or without VSV at MOI = 10 for eight hours, after which cell extracts were prepared with lysis buffer (20 mM Tris-HCl pH 7.5, 150 mM NaCl, 1 mM EDTA, 10% glycerol, 1% Nonidet P-40, 30 mM NaF, 5 mM Na_3_VO_4_, 20 mM iodoacetamide, and 2 mM phenylmethylsulfonyl fluoride). Immunoprecipitation used an anti-RIG-I Rabbit monoclonal antibody (D14G6, Cell Signaling Technology). To detect endogenous K63-linked polyubiquitin chain that is ligated to RIG-I, 6×10^7^ of mouse splenocyte were infected with SeV at MOI = 0.2 for 24 hours. Immunoprecipitation was performed with anti-RIG-I mAb (D14G6). Anti-K63-linkage specific polyubiquitin (D7A11) Rabbit mAb (Cell Signaling) was used for western blotting. HEK293FT cells were transfected with or without 0.8 µg of HCV dsRNA in a 6-well plate. HCV dsRNA (HCV 3′ UTR polyU/UC region) was synthesized using T7 and SP6 RNA polymerase as previously described [46]. Cell lysates were prepared at the indicated times. Immunoprecipitation was performed with an anti-RIG-I mouse monoclonal antibody (Alme-1). An anti-FLAG M2 monoclonal antibody (Sigma) was used for the immunoprecipitation of FLAG-tagged protein. An anti-TRIM25 rabbit polyclonal antibody (abcam), an anti-p-TBK1 rabbit mAb (Cell Signaling Technology), an anti-NAK (TBK1) rabbit mAb (EP611Y), and an anti-RNF135 (Riplet) pAb (SIGMA), were used for western blotting. For ubiquitination assay, immunoprecipitates were washed three times with high salt lysis buffer ((20 mM Tris-HCl pH 7.5, 1M NaCl, 1 mM EDTA, 10% glycerol, 1% Nonidet P-40, 30 mM NaF, 5 mM Na_3_VO_4_, 20 mM iodoacetamide, and 2 mM phenylmethylsulfonyl fluoride) to dissociate unanchored polyubiquitin chain [21], and then washed once with normal lysis buffer described above for SDS-PAG analysis. Band intensity was semi-quantified using Photoshop software.

### RNAi

siRNAs for human Riplet (Silencer Select Validated siRNA) and negative control were purchased from Ambion. siRNA sequences for Riplet are: (sense) GGA ACA UCU UGU AGA CAU Utt and (anti-sense) AAU GUC UAC AAG AUG UUC CCac. siRNA was transfected into cells using RNAiMax Reagent (Invitrogen) according to the manufacture's instructions.

### In vitro NS3/4A cleavage assay

FLAG-tagged Riplet was expressed in HEK293FT cells, and cell lysate was prepared with the lysis buffer described above. The protein was immunoprecipitated with anti-FLAG antibody and protein G sepharose beads, and washed with Buffer B (20 mM Tris-HCl pH 7.5, 150 mM NaCl, 10% glycerol, 1% Nonidet P-40). The samples were suspended in 50 µl of Buffer B, and incubated with 400 ng of recombinant NS3-4A (rNS3-4A) protein at 37°C for one hour, and then subjected to SDS-PAGE analysis. The NS3-4A protein was purchased from AnaSpec Inc (CA). N-terminal GST-fused Riplet (1–210 aa) (rRiplet) was purchased from Abnova. 500 ng of rRiplet was incubated with or without 500 ng of rNS3-4A in 10 µl of reaction buffer (20 mM Tris-HCl (7.5), 4% Glycerol, 5 mM DTT, 150 mM NaCl, 0.1% of Triton-X100, 0.9% polyvinyl alcohol) at 37°C for 30 min.

### Accession numbers

The accession numbers are Riplet (BAG84604), TRIM25 (NP_005073), TBK1 (NP_037386), IKK-ε (AAF45307), IPS-1 (BAE79738), RIG-I (NP_055129), and G3BP (CAG38772).

## Supporting Information

Figure S1 **K63-linked polyubiquitination of RIG-I RD.** HA-tagged ubiquitin and FLAG-tagged RIG-I RD expression vectors were transfected into HEK293FT cells. 24 hours after transfection, the cells were infected with VSV at MOI = 1 for six hours. Then, cell lysate was prepared. Immunoprecipitation was carried out using anti-FLAG antibody. The samples were subjected to SDS-PAGE, and the proteins were detected by western blotting using anti-HA, FLAG, and K63-linked polyubiquitin specific antibodies.
(TIF)

Figure S2 **Intracellular localization of RIG-I, NEMO, and p-TBK1 proteins.** (A) HeLa cells were transfected with HCV dsRNA using lipofectamine 2000 reagent. The cells were fixed six hours after transfection. The microscopic analysis was performed using anti-RIG-I mAb (Alme-1) and anti-NEMO pAb. (B) HeLa cells were transfected with HCV dsRNA using lipofectamine 2000 reagent (Invitrogen). The cells were fixed at indicated hour. The microscopic analysis was performed using anti-RIG-I mAb (Alme-1). (C) HepG2 cells were transfected with HCV dsRNA using lipofectamine 200 reagent. The cells were fixed six hours after the transfection. The microscopic analysis was performed using anti-RIG-I (Alme-1) mAb and anti-p-TBK1 mAb.
(TIF)

Figure S3 **NS3-4A of HCV cleaves IPS-1 and Riplet but not IKK-ε.** (A) HA-tagged Riplet was transfected into HEK293 cells together with NS3-4A. 24 hours after transfection, cell lysate was prepared and subjected to SDS-PAGE. The proteins were detected by western blotting and CBB staining. (B, C) HA-tagged IKK-ε (B) or IPS-1 (C) expression vectors were transfected into HEK293FT cells with or without NS3-4A of HCV expression vector. 24 hours after the transfection, the cell lysate was prepared, and analyzed by SDS-PAGE. The proteins were detected by western blotting using anti-HA or anti-β actin antibodies. (D) HA-tagged IPS-1 or HA-tagged Riplet expression vector was transfected into HEK293FT cells with or without NS3-4A expression vectors. 24 hours after transfection, cell lysate was prepared and subjected to SDS-PAGE. The proteins were detected by western blotting using anti-HA antibody. (E, F) N-terminal FLAG-tagged Riplet (E) or C-terminal HA-tagged Riplet (F) expression vector was transfected into HEK293FT cells with NS3-4A or NS3-4A*. 24 hours after the transfection, cell lysates were analyzed by SDS-PAGE. (G) HA-tagged wild-type Riplet or mutant Riplet-C21A expression vector were transfected into HEK293FT cells with NS3-4A or NS3-4A*. 24 hours after the transfection, the cell lysate was prepared, and analyzed by SDS-PAGE. The proteins were detected by western blotting using anti-HA or anti-β actin antibodies. (H, I) RIG-I, Riplet, Riplet-3A (H), and/or Riplet C21A (I) mutant expression vectors were transfected into HEK293 cells together with p125luc reporter and *Renilla* luciferase. 24 hours after transfection, luciferase activity was measured.
(TIF)

Figure S4 **siRNA for Riplet or control was transfected into HeLa cells in 24-well plate using RNAi MAX (Invitrogen) according to manufacture's protocol.** 48 hours after transfection, the cells were transfected with 100 ng of HCV dsRNA. Six hours after transfection, the cells were fixed and stained with anti-RIG-I mAb (Alme-1) and anti-mouse Alexa-488 Ab.
(TIF)

## References

[1] Kato H, Takeuchi O, Sato S, Yoneyama M, Yamamoto M, et al (2006) Differential roles of MDA5 and RIG-I helicases in the recognition of RNA viruses. Nature 441: 101–105. 16625202 10.1038/nature04734

[2] Yoneyama M, Kikuchi M, Natsukawa T, Shinobu N, Imaizumi T, et al (2004) The RNA helicase RIG-I has an essential function in double-stranded RNA-induced innate antiviral responses. Nat Immunol 5: 730–737. 15208624 10.1038/ni1087

[3] Saito T, Hirai R, Loo YM, Owen D, Johnson CL, et al (2007) Regulation of innate antiviral defenses through a shared repressor domain in RIG-I and LGP2. Proc Natl Acad Sci U S A 104: 582–587. 17190814 10.1073/pnas.0606699104 PMC1766428

[4] Kowalinski E, Lunardi T, McCarthy AA, Louber J, Brunel J, et al (2011) Structural basis for the activation of innate immune pattern-recognition receptor RIG-I by viral RNA. Cell 147: 423–435. 22000019 10.1016/j.cell.2011.09.039

[5] Xu LG, Wang YY, Han KJ, Li LY, Zhai Z, et al (2005) VISA is an adapter protein required for virus-triggered IFN-beta signaling. Mol Cell 19: 727–740. 16153868 10.1016/j.molcel.2005.08.014

[6] Seth RB, Sun L, Ea CK, Chen ZJ (2005) Identification and characterization of MAVS, a mitochondrial antiviral signaling protein that activates NF-kappaB and IRF 3. Cell 122: 669–682. 16125763 10.1016/j.cell.2005.08.012

[7] Meylan E, Curran J, Hofmann K, Moradpour D, Binder M, et al (2005) Cardif is an adaptor protein in the RIG-I antiviral pathway and is targeted by hepatitis C virus. Nature 437: 1167–1172. 16177806 10.1038/nature04193

[8] Kawai T, Takahashi K, Sato S, Coban C, Kumar H, et al (2005) IPS-1, an adaptor triggering RIG-I- and Mda5-mediated type I interferon induction. Nat Immunol 6: 981–988. 16127453 10.1038/ni1243

[9] Zhao T, Yang L, Sun Q, Arguello M, Ballard DW, et al (2007) The NEMO adaptor bridges the nuclear factor-kappaB and interferon regulatory factor signaling pathways. Nat Immunol 8: 592–600. 17468758 10.1038/ni1465

[10] McWhirter SM, Fitzgerald KA, Rosains J, Rowe DC, Golenbock DT, et al (2004) IFN-regulatory factor 3-dependent gene expression is defective in Tbk1-deficient mouse embryonic fibroblasts. Proc Natl Acad Sci U S A 101: 233–238. 14679297 10.1073/pnas.2237236100 PMC314168

[11] Hemmi H, Takeuchi O, Sato S, Yamamoto M, Kaisho T, et al (2004) The roles of two IkappaB kinase-related kinases in lipopolysaccharide and double stranded RNA signaling and viral infection. J Exp Med 199: 1641–1650. 15210742 10.1084/jem.20040520 PMC2212809

[12] Lo YC, Lin SC, Rospigliosi CC, Conze DB, Wu CJ, et al (2009) Structural basis for recognition of diubiquitins by NEMO. Mol Cell 33: 602–615. 19185524 10.1016/j.molcel.2009.01.012 PMC2749619

[13] Fitzgerald KA, McWhirter SM, Faia KL, Rowe DC, Latz E, et al (2003) IKKepsilon and TBK1 are essential components of the IRF3 signaling pathway. Nat Immunol 4: 491–496. 12692549 10.1038/ni921

[14] Oshiumi H, Matsumoto M, Seya T (2012) Ubiquitin-mediated modulation of the cytoplasmic viral RNA sensor RIG-I. J Biochem 151: 5–11. 21890623 10.1093/jb/mvr111

[15] Gack MU, Shin YC, Joo CH, Urano T, Liang C, et al (2007) TRIM25 RING-finger E3 ubiquitin ligase is essential for RIG-I-mediated antiviral activity. Nature 446: 916–920. 17392790 10.1038/nature05732

[16] Liu HM, Loo YM, Horner SM, Zornetzer GA, Katze MG, et al (2012) The Mitochondrial Targeting Chaperone 14-3-3epsilon Regulates a RIG-I Translocon that Mediates Membrane Association and Innate Antiviral Immunity. Cell Host Microbe 11: 528–537. 22607805 10.1016/j.chom.2012.04.006 PMC3358705

[17] Arnaud N, Dabo S, Akazawa D, Fukasawa M, Shinkai-Ouchi F, et al (2011) Hepatitis C virus reveals a novel early control in acute immune response. PLoS Pathog 7: e1002289. 22022264 10.1371/journal.ppat.1002289 PMC3192838

[18] Jiang X, Kinch LN, Brautigam CA, Chen X, Du F, et al (2012) Ubiquitin-Induced Oligomerization of the RNA Sensors RIG-I and MDA5 Activates Antiviral Innate Immune Response. Immunity 36: 959–973. 22705106 10.1016/j.immuni.2012.03.022 PMC3412146

[19] Zeng W, Sun L, Jiang X, Chen X, Hou F, et al (2010) Reconstitution of the RIG-I pathway reveals a signaling role of unanchored polyubiquitin chains in innate immunity. Cell 141: 315–330. 20403326 10.1016/j.cell.2010.03.029 PMC2919214

[20] Friedman CS, O'Donnell MA, Legarda-Addison D, Ng A, Cardenas WB, et al (2008) The tumour suppressor CYLD is a negative regulator of RIG-I-mediated antiviral response. EMBO Rep 9: 930–936. 18636086 10.1038/embor.2008.136 PMC2529351

[21] Oshiumi H, Matsumoto M, Hatakeyama S, Seya T (2009) Riplet/RNF135, a RING finger protein, ubiquitinates RIG-I to promote interferon-beta induction during the early phase of viral infection. J Biol Chem 284: 807–817. 19017631 10.1074/jbc.M804259200

[22] Gao D, Yang YK, Wang RP, Zhou X, Diao FC, et al (2009) REUL is a novel E3 ubiquitin ligase and stimulator of retinoic-acid-inducible gene-I. PLoS One 4: e5760. 19484123 10.1371/journal.pone.0005760 PMC2684588

[23] Oshiumi H, Miyashita M, Inoue N, Okabe M, Matsumoto M, et al (2010) The ubiquitin ligase Riplet is essential for RIG-I-dependent innate immune responses to RNA virus infection. Cell Host Microbe 8: 496–509. 21147464 10.1016/j.chom.2010.11.008

[24] Saito T, Owen DM, Jiang F, Marcotrigiano J, Gale M Jr (2008) Innate immunity induced by composition-dependent RIG-I recognition of hepatitis C virus RNA. Nature 454: 523–527. 18548002 10.1038/nature07106 PMC2856441

[25] Foy E, Li K, Wang C, Sumpter R Jr, Ikeda M, et al (2003) Regulation of interferon regulatory factor-3 by the hepatitis C virus serine protease. Science 300: 1145–1148. 12702807 10.1126/science.1082604

[26] Li XD, Sun L, Seth RB, Pineda G, Chen ZJ (2005) Hepatitis C virus protease NS3/4A cleaves mitochondrial antiviral signaling protein off the mitochondria to evade innate immunity. Proc Natl Acad Sci U S A 102: 17717–17722. 16301520 10.1073/pnas.0508531102 PMC1308909

[27] Ebihara T, Shingai M, Matsumoto M, Wakita T, Seya T (2008) Hepatitis C virus-infected hepatocytes extrinsically modulate dendritic cell maturation to activate T cells and natural killer cells. Hepatology 48: 48–58. 18537195 10.1002/hep.22337

[28] Li K, Foy E, Ferreon JC, Nakamura M, Ferreon AC, et al (2005) Immune evasion by hepatitis C virus NS3/4A protease-mediated cleavage of the Toll-like receptor 3 adaptor protein TRIF. Proc Natl Acad Sci U S A 102: 2992–2997. 15710891 10.1073/pnas.0408824102 PMC548795

[29] Arimoto K, Takahashi H, Hishiki T, Konishi H, Fujita T, et al (2007) Negative regulation of the RIG-I signaling by the ubiquitin ligase RNF125. Proc Natl Acad Sci U S A 104: 7500–7505. 17460044 10.1073/pnas.0611551104 PMC1863485

[30] Onomoto K, Jogi M, Yoo JS, Narita R, Morimoto S, et al (2012) Critical role of an antiviral stress granule containing RIG-I and PKR in viral detection and innate immunity. PLoS One 7: e43031. 22912779 10.1371/journal.pone.0043031 PMC3418241

[31] Kageyama M, Takahasi K, Narita R, Hirai R, Yoneyama M, et al (2011) 55 Amino acid linker between helicase and carboxyl terminal domains of RIG-I functions as a critical repression domain and determines inter-domain conformation. Biochem Biophys Res Commun 415: 75–81. 22020100 10.1016/j.bbrc.2011.10.015

[32] Rajsbaum R, Albrecht RA, Wang MK, Maharaj NP, Versteeg GA, et al (2012) Species-Specific Inhibition of RIG-I Ubiquitination and IFN Induction by the Influenza A Virus NS1 Protein. PLoS Pathog 8: e1003059. 23209422 10.1371/journal.ppat.1003059 PMC3510253

[33] Soulat D, Burckstummer T, Westermayer S, Goncalves A, Bauch A, et al (2008) The DEAD-box helicase DDX3X is a critical component of the TANK-binding kinase 1-dependent innate immune response. EMBO J 27: 2135–2146. 18583960 10.1038/emboj.2008.126 PMC2453059

[34] Gack MU, Albrecht RA, Urano T, Inn KS, Huang IC, et al (2009) Influenza A virus NS1 targets the ubiquitin ligase TRIM25 to evade recognition by the host viral RNA sensor RIG-I. Cell Host Microbe 5: 439–449. 19454348 10.1016/j.chom.2009.04.006 PMC2737813

[35] Bartenschlager R, Ahlborn-Laake L, Yasargil K, Mous J, Jacobsen H (1995) Substrate determinants for cleavage in cis and in trans by the hepatitis C virus NS3 proteinase. J Virol 69: 198–205. 7983710 10.1128/jvi.69.1.198-205.1995 PMC188564

[36] Borden KL, Freemont PS (1996) The RING finger domain: a recent example of a sequence-structure family. Curr Opin Struct Biol 6: 395–401. 8804826 10.1016/s0959-440x(96)80060-1

[37] Aly HH, Oshiumi H, Shime H, Matsumoto M, Wakita T, et al (2011) Development of mouse hepatocyte lines permissive for hepatitis C virus (HCV). PLoS One 6: e21284. 21731692 10.1371/journal.pone.0021284 PMC3120852

[38] Gack MU, Kirchhofer A, Shin YC, Inn KS, Liang C, et al (2008) Roles of RIG-I N-terminal tandem CARD and splice variant in TRIM25-mediated antiviral signal transduction. Proc Natl Acad Sci U S A 105: 16743–16748. 18948594 10.1073/pnas.0804947105 PMC2575490

[39] Takahasi K, Yoneyama M, Nishihori T, Hirai R, Kumeta H, et al (2008) Nonself RNA-sensing mechanism of RIG-I helicase and activation of antiviral immune responses. Mol Cell 29: 428–440. 18242112 10.1016/j.molcel.2007.11.028

[40] Paz S, Vilasco M, Arguello M, Sun Q, Lacoste J, et al (2009) Ubiquitin-regulated recruitment of IkappaB kinase epsilon to the MAVS interferon signaling adapter. Mol Cell Biol 29: 3401–3412. 19380491 10.1128/MCB.00880-08 PMC2698723

[41] Zeng W, Xu M, Liu S, Sun L, Chen ZJ (2009) Key role of Ubc5 and lysine-63 polyubiquitination in viral activation of IRF3. Mol Cell 36: 315–325. 19854139 10.1016/j.molcel.2009.09.037 PMC2779157

[42] Oganesyan G, Saha SK, Guo B, He JQ, Shahangian A, et al (2006) Critical role of TRAF3 in the Toll-like receptor-dependent and -independent antiviral response. Nature 439: 208–211. 16306936 10.1038/nature04374

[43] Suzuki T, Ishii K, Aizaki H, Wakita T (2007) Hepatitis C viral life cycle. Adv Drug Deliv Rev 59: 1200–1212. 17825945 10.1016/j.addr.2007.04.014

[44] Ikeda M, Abe K, Dansako H, Nakamura T, Naka K, et al (2005) Efficient replication of a full-length hepatitis C virus genome, strain O, in cell culture, and development of a luciferase reporter system. Biochem Biophys Res Commun 329: 1350–1359. 15766575 10.1016/j.bbrc.2005.02.138

[45] Oshiumi H, Okamoto M, Fujii K, Kawanishi T, Matsumoto M, et al (2011) The TLR3/TICAM-1 pathway is mandatory for innate immune responses to poliovirus infection. J Immunol 187: 5320–5327. 21998457 10.4049/jimmunol.1101503

[46] Oshiumi H, Ikeda M, Matsumoto M, Watanabe A, Takeuchi O, et al (2010) Hepatitis C virus core protein abrogates the DDX3 function that enhances IPS-1-mediated IFN-beta induction. PLoS One 5: e14258. 21170385 10.1371/journal.pone.0014258 PMC2999533

