# Supplementary figures and images for: A Distinct Role of Riplet-Mediated K63-Linked Polyubiquitination of the RIG-I Repressor Domain in Human Antiviral Innate Immune Responses
Source: PLoS Pathog. 2013 Aug 8;9(8):e1003533. doi: 10.1371/journal.ppat.1003533 (PMC3738492; doi:10.1371/journal.ppat.1003533)

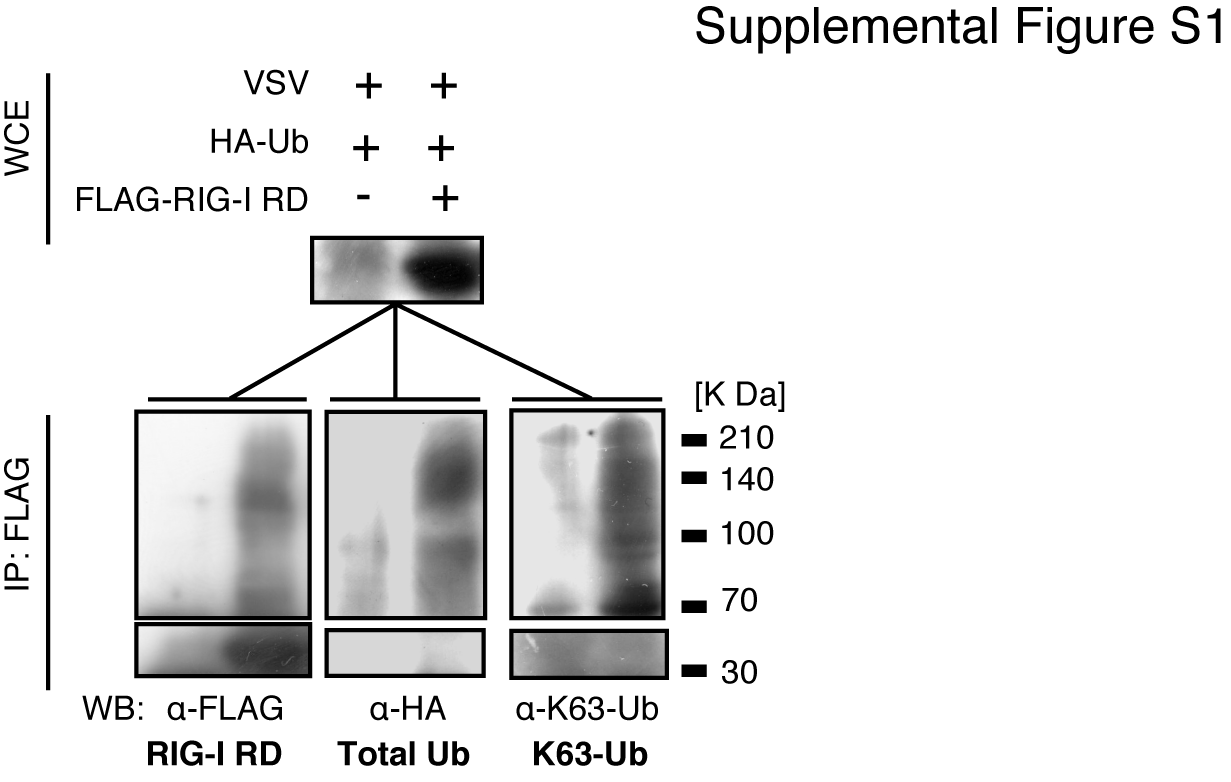

Supplement: Figure S1 — K63-linked polyubiquitination of RIG-I RD. HA-tagged ubiquitin and FLAG-tagged RIG-I RD expression vectors were transfected into HEK293FT cells. 24 hours after transfection, the cells were infected with VSV at MOI = 1 for six hours. Then, cell lysate was prepared. Immunoprecipitation was carried out using anti-FLAG antibody. The samples were subjected to SDS-PAGE, and the proteins were detected by western blotting using anti-HA, FLAG, and K63-linked polyubiquitin specific antibodies. (TIF) [file ppat.1003533.s001.tif]

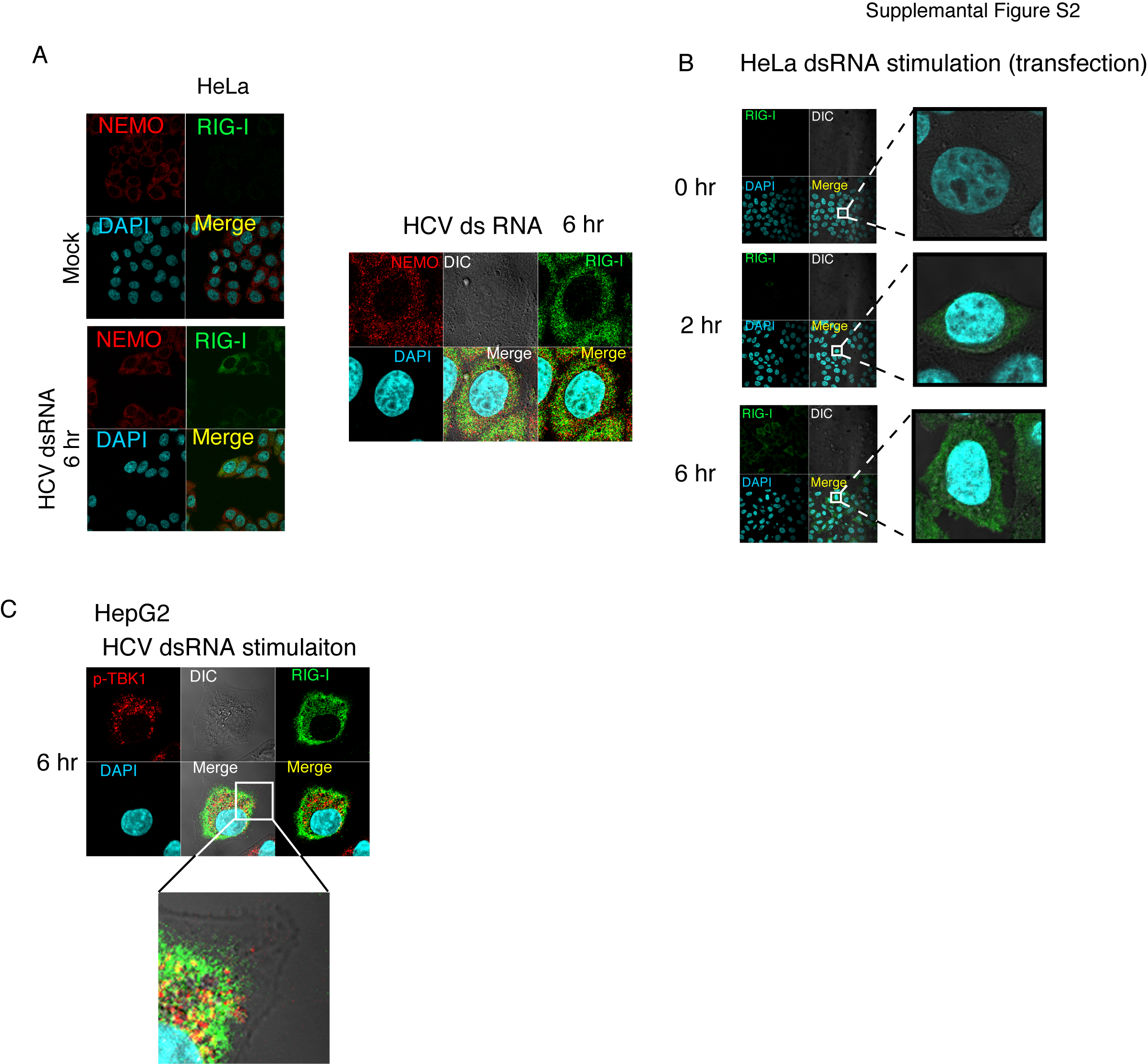

Supplement: Figure S2 — Intracellular localization of RIG-I, NEMO, and p-TBK1 proteins. (A) HeLa cells were transfected with HCV dsRNA using lipofectamine 2000 reagent. The cells were fixed six hours after transfection. The microscopic analysis was performed using anti-RIG-I mAb (Alme-1) and anti-NEMO pAb. (B) HeLa cells were transfected with HCV dsRNA using lipofectamine 2000 reagent (Invitrogen). The cells were fixed at indicated hour. The microscopic analysis was performed using anti-RIG-I mAb (Alme-1). (C) HepG2 cells were transfected with HCV dsRNA using lipofectamine 200 reagent. The cells were fixed six hours after the transfection. The microscopic analysis was performed using anti-RIG-I (Alme-1) mAb and anti-p-TBK1 mAb. (TIF) [file ppat.1003533.s002.tif]

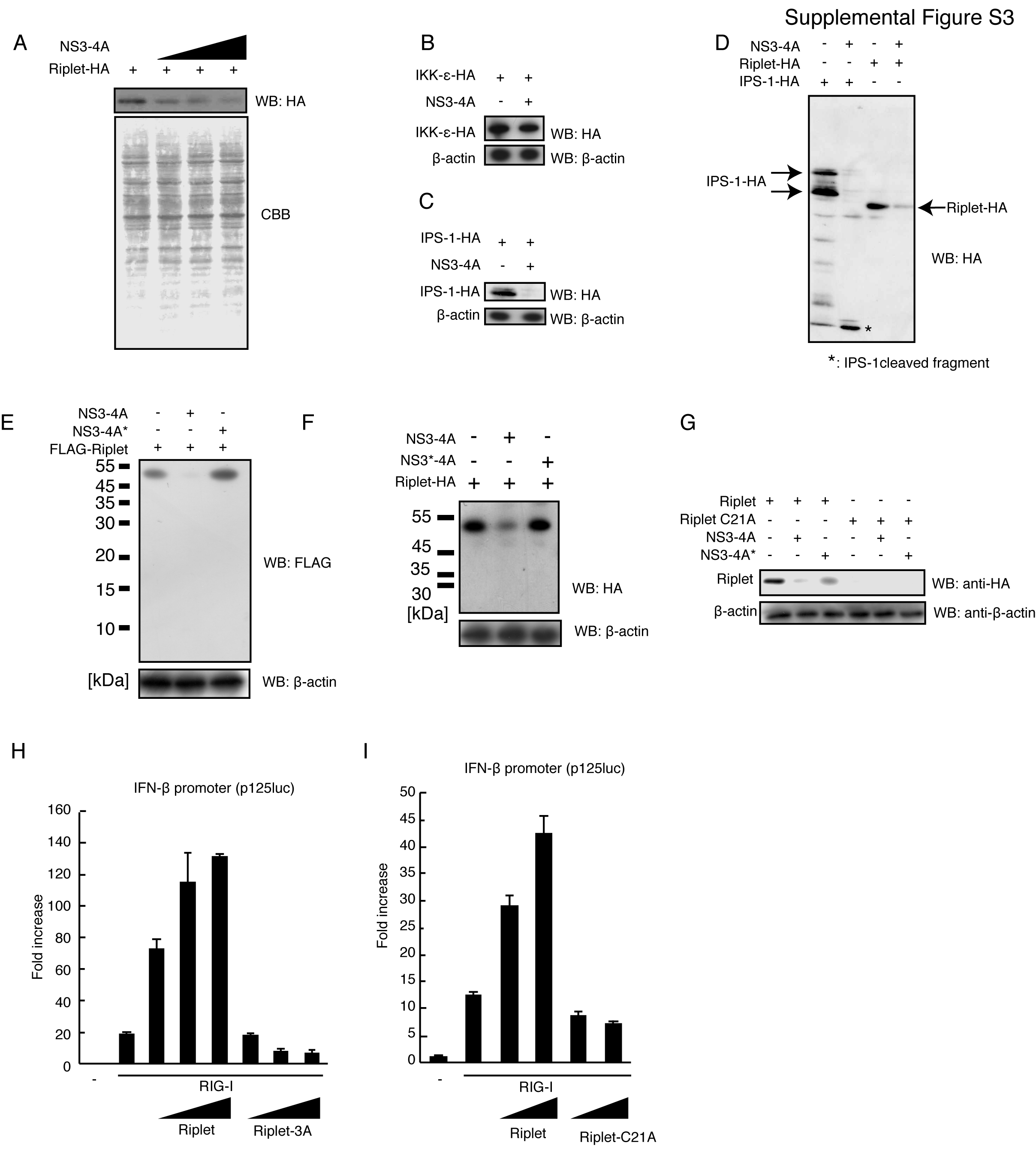

Supplement: Figure S3 — NS3-4A of HCV cleaves IPS-1 and Riplet but not IKK-ε. (A) HA-tagged Riplet was transfected into HEK293 cells together with NS3-4A. 24 hours after transfection, cell lysate was prepared and subjected to SDS-PAGE. The proteins were detected by western blotting and CBB staining. (B, C) HA-tagged IKK-ε (B) or IPS-1 (C) expression vectors were transfected into HEK293FT cells with or without NS3-4A of HCV expression vector. 24 hours after the transfection, the cell lysate was prepared, and analyzed by SDS-PAGE. The proteins were detected by western blotting using anti-HA or anti-β actin antibodies. (D) HA-tagged IPS-1 or HA-tagged Riplet expression vector was transfected into HEK293FT cells with or without NS3-4A expression vectors. 24 hours after transfection, cell lysate was prepared and subjected to SDS-PAGE. The proteins were detected by western blotting using anti-HA antibody. (E, F) N-terminal FLAG-tagged Riplet (E) or C-terminal HA-tagged Riplet (F) expression vector was transfected into HEK293FT cells with NS3-4A or NS3-4A*. 24 hours after the transfection, cell lysates were analyzed by SDS-PAGE. (G) HA-tagged wild-type Riplet or mutant Riplet-C21A expression vector were transfected into HEK293FT cells with NS3-4A or NS3-4A*. 24 hours after the transfection, the cell lysate was prepared, and analyzed by SDS-PAGE. The proteins were detected by western blotting using anti-HA or anti-β actin antibodies. (H, I) RIG-I, Riplet, Riplet-3A (H), and/or Riplet C21A (I) mutant expression vectors were transfected into HEK293 cells together with p125luc reporter and Renilla luciferase. 24 hours after transfection, luciferase activity was measured. (TIF) [file ppat.1003533.s003.tif]

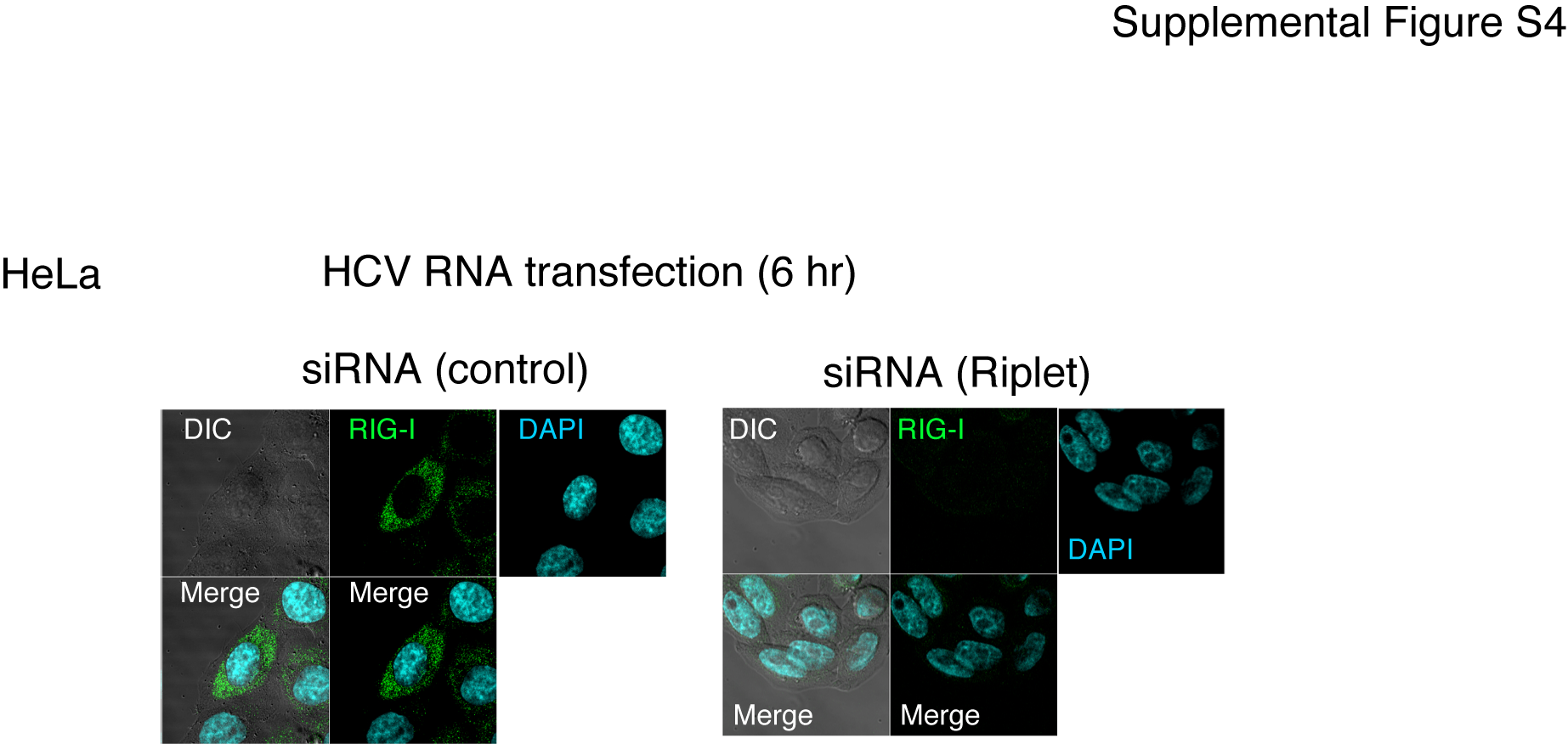

Supplement: Figure S4 — siRNA for Riplet or control was transfected into HeLa cells in 24-well plate using RNAi MAX (Invitrogen) according to manufacture's protocol. 48 hours after transfection, the cells were transfected with 100 ng of HCV dsRNA. Six hours after transfection, the cells were fixed and stained with anti-RIG-I mAb (Alme-1) and anti-mouse Alexa-488 Ab. (TIF) [file ppat.1003533.s004.tif]
